# Marine Longilenes, Oxasqualenoids with Ser-Thr Protein Phosphatase 2A Inhibition Activity

**DOI:** 10.3390/md16040131

**Published:** 2018-04-17

**Authors:** Francisco Cen-Pacheco, Claudia Pérez Manríquez, María Luisa Souto, Manuel Norte, José Javier Fernández, Antonio Hernández Daranas

**Affiliations:** 1 Instituto Universitario de Bio-Orgánica Antonio González (IUBO AG), Centro de Investigaciones Biomédicas de Canarias (CIBICAN), Universidad de La Laguna (ULL), Avenida Astrofísico Francisco Sánchez 2, 38206 Tenerife, Spain; claudiaperez@udec.cl (C.P.M.); msouto@ull.edu.es (M.L.S.); mnorte@ull.edu.es (M.N.); 2 Facultad de Bioanálisis, Campus-Veracruz, Universidad Veracruzana, Veracruz 91700, Mexico; fcen@uv.mx; 3 Departamento de Botánica, Facultad de Ciencias Naturales y Oceanográficas, Universidad de Concepción, Barrio Universitario, Concepción, Región del Biobío 4030000, Chile; 4 Departamento de Química Orgánica, Universidad de La Laguna (ULL), Avenida Astrofísico Francisco Sánchez 2, 38206 Tenerife, Spain; 5 Instituto de Productos Naturales y Agrobiología (IPNA), Consejo Superior de Investigaciones Científicas (CSIC), Avenida Astrofísico Francisco Sánchez 2, 38206 Tenerife, Spain

**Keywords:** phosphatase inhibition, marine polyether, longilenes, docking simulation, red alga, *Laurencia viridis*

## Abstract

The red seaweed *Laurencia viridis* is a rich source of oxygenated secondary metabolites that were derived from squalene. We report here the structures of three novel compounds, (+)-longilene peroxide (**1**), longilene (**2**), and (+)-prelongilene (**3**) that were isolated from this alga, in addition to other substances, **4** and **5**, resulting from their acid-mediated degradation. The effect of compounds **1** and **3** against Ser-Thr protein phosphatase type 2A (PP2A) was evaluated, showing that (+)-longilene peroxide (**1**) inhibited PP2A (IC_50_ 11.3 μM). In order to explain the interaction between PP2A and compounds **1** and **3**, molecular docking simulations onto the PP2A enzyme-binding region were used.

## 1. Introduction

The phosphorylation and dephosphorylation processes that were controlled by protein kinases and phosphatases are essential for the appropriate functioning of all living cells [1]. However, while kinases were established as drug targets several years ago, their counterpart phosphatases have only been considered recently. In particular, Ser-Thr protein phosphatases type 1 (PP1) and 2A (PP2A) are involved in the control of the cell cycle, apoptosis, signal transduction, metabolism, protein synthesis, as well as tumour promoters and suppressors [2,3]. Therefore, inhibitors of these enzymes have been investigated as a potential novel therapeutic approach in diseases, such as cancer, osteoporosis, diabetes, and Alzheimer’s. disease [4,5]. A number of marine natural products have been identified as inhibitors of Ser-Thr protein phosphatases. The first example was okadaic acid—which was isolated in 1981 from the marine sponge *Halicondria okadai*—a natural toxin that is known to cause diarrhetic shellfish poisoning [6]. Other protein phosphatase inhibitors include cyclic peptides, such as microcystin-LR and motuporin, polyketides, such as spirastrellolide A, tautomycin, and calliculin A and C, and terpenes, such as thyrsiferyl-23-acetate and cantharidin [4,7,8].

**Figure 1:**
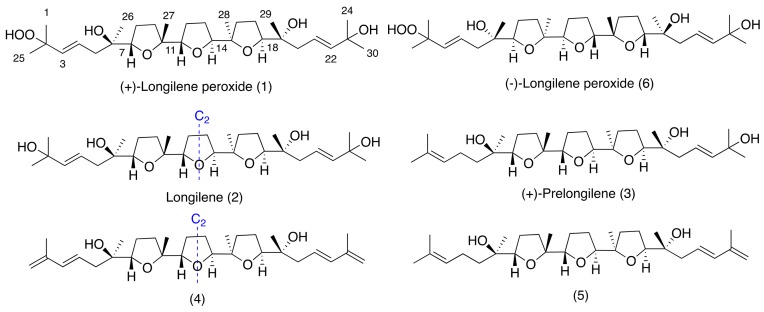
Structures of longilene metabolites isolated from *Laurencia viridis.*

Squalene-derived polyethers belonging to the thyrsiferol and venustatriol series have shown different biological activities, such as cytotoxicity, integrin antagonist activity, and protein phosphate type 2A inhibition [9,10,11]. As part of our continuing interest in new bioactive molecules from marine sources, we report herein the isolation and the structural elucidation of three new marine polyether natural products, (+)-longilene peroxide (**1**), longilene (**2**), and (+)-prelongilene (**3**), and another two compounds (**4**–**5**), which were derived from the rapid degradation of **2** and **3** (Figure 1). Additionally, the PP2A inhibitory effect of the less labile compounds (+)-longilene peroxide (**1**) and (+)-prelongilene (**3**) was tested, and their likely binding conformations proposed on the basis of molecular docking simulations.

## 2. Results and Discussion

**Figure 2:**
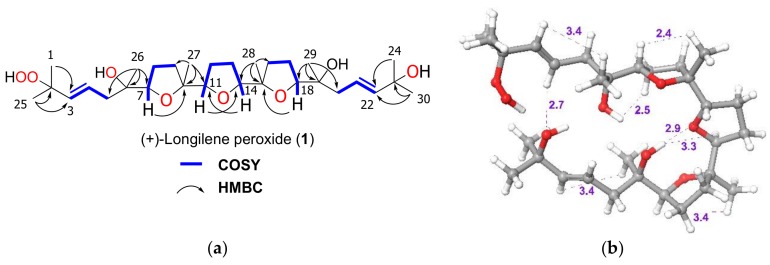
(**a**) Selected NMR-derived correlations HMBC and COSY and (**b**) perspective view with significant ROESY relationships (dashed line and distances in Ångstrom) observed for (+)-longilene peroxide (**1**).

The molecular formula of (+)-longilene peroxide (**1**) C_30_H_52_O_8_Na, was established by HRESIMS analysis, where its sodiated molecular ion was observed at *m*/*z* 563.3571, calculated 563.3559, [M + Na]^+^. Analysis of the two-dimensional (2D) NMR spectra (COSY, HSQC, HMBC, and ROESY) of **1** indicated the presence of eight methyl, eight methylene and eight methine groups (four of the latter on sp^2^ carbons), in addition to six oxygenated quaternary carbons. Analysis of such spectroscopic and spectrometric data (Table 1) suggested that the structure of **1** was identical to that of (−)-longilene peroxide (**6**), as reported by Itokawa et al. in 1991 [12], isolated from the methanol extract of the *Eurycoma longifolia* wood (Simaroubaceae) (Figure 1 and Figure 2).

The stereostructure and the conformation of the terrestrial molecule was assigned on the basis of X-ray crystallographic analysis, although the absolute stereochemistry was not defined. Afterward, Morimoto et al. solved this problem by means of an elegant total synthesis approach [13]. The specific rotation value that was measured for the marine triterpene **1** was ${\left[\alpha \right]}_{D}^{25}$ +47, as opposed to ${\left[\alpha \right]}_{D}^{25}$ −23, reported for the metabolite isolated from *Eurycoma longifolia*, and ${\left[\alpha \right]}_{D}^{27}$ −45 for the synthetic substance **6**. According to these value, the marine polyether that was isolated from *Laurencia viridis* must be the enantiomeric form of the terrestrial (−)-longilene peroxide (**6**) as shown in Figure 1. Therefore, the absolute configuration of **1** should be 6*R*, 7*S*, 10*R*, 11*S*, 14*S*, 15*R*, 18*S*, and 19*R*.

**Figure 3:**
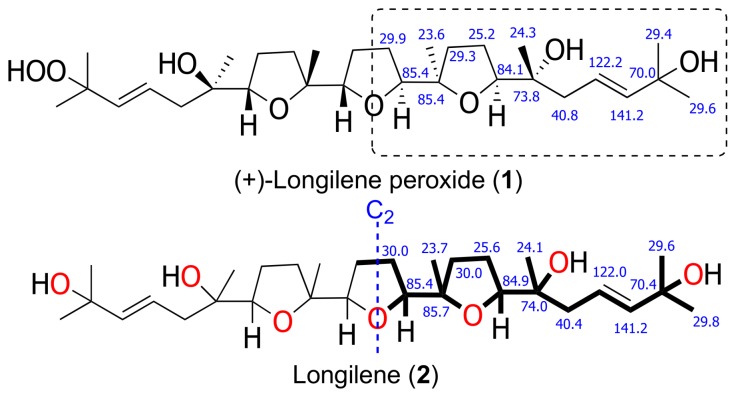
Selected ^13^C NMR chemical shift comparison between similar fragment in (+)-longilene peroxide (**1**) and longilene (**2**) (bold line).

The HRESIMS analysis of the very unstable metabolite longilene (**2**) provided the molecular formula C_30_H_52_O_7_ that was based on the *m/z* 524.3716 (calculated for 524.3713 [M]^+^), as observed. However, its spectroscopic data revealed only half of the expected signals in both the ^1^H and ^13^C NMR spectra, suggesting the presence of a Cs symmetry element in this metabolite. Further study of the spectroscopic and spectrometric data of **2** permitted a full NMR assignment. In addition, the equivalence of the observed ^13^C chemical shifts at C-2 and C-23 confirmed the presence of a hydroxyl group at C-2 in **2**, rather than the hydroperoxide functional group in compound **1**. The resemblance of the NMR data in **1** and **2** suggested that the relative configurations of the stereocentres in **2** match those that were previously described for (+)-longilene peroxide (**1**) in accordance with a C_2_ symmetry (Table 1 and Figure 3).

Compound **3**, (+)-prelongilene, was obtained as an amorphous white solid. Its molecular formula was established by ESI-HRMS as C_30_H_52_O_6_ (*m/z* 531.3655; calculated for 531.3662, [M + Na]^+^). The structure of this metabolite was determined by comparison of its spectroscopic data with those of (+)-longilene peroxide (**1**) (Table 1 and Table 2). Thus, the main difference in their NMR spectra was in the C-1→C-5 fragment. A ^1^H-^1^H spin system was built for this fragment, starting from the proton signal H-3 (δ_H_ 5.08, t, *J* = 6.9 Hz), which was coupled with the methylene H_2_-4 (δ_H_ 1.97/2.04), and this sequentially connected with the diastereotopic protons H_2_-5 (δ_H_ 1.29/1.43). Long-range ^1^H-^13^C connectivities that were extracted from the HMBC experiment allowed us to connect this substructure within the molecule. Thus, the planar structure of **3** was established by correlations from the geminal methyls H_3_-1/H_3_-25 (δ_H_ 1.60/1.66) to C-2 (δ_C_ 131.1) and C-3 (δ_C_ 124.8), and those that were observed from the protons H_2_-5, H_3_-26 (δ_H_ 1.27) and H-7 (δ_H_ 3.73, dd, *J* = 6.9 and 7.6 Hz) to the quaternary oxygen-bearing carbon C-6 (δ_C_ 72.5). The relative stereochemistry we propose here derives from comparison of the spectroscopical data of **3** (chemical shift and ROESY correlations) with those of **1** and **2**. Additionally it seems likely that this compound is a precursor of (+)-longilene peroxide (**1**), by action of peroxidase enzymes. This would follow a similar pathway to that observed in the biogenesis of datylomelanes in the *Laurencia* genus [14].

Together with the previously described metabolites, compounds **4** and **5** (Tables S4 and S5) were characterized as degradation by products of **2** and **3**. Thus, the origin of **4** and **5** was explained by dehydration of the terminal alcohols present in **2** and **3**, respectively. Briefly, when compound **2** was dissolved in CDCl_3_, we observed its total conversion into **4** after 48 h (see Supplementary Materials). Its NMR data confirmed the presence of conjugated double bonds in the C-1→C-4 and C-21→C-24 portions of **4**. To confirm this hypothesis, the conjugated double bonds in the C-21→C-24 fragment were obtained in acid media by treatment of **3** with Dowex-50 in CDCl_3_, to obtain compound **5** (see Supplementary Materials).

Some marine polyethers derived from squalene show selective inhibition of protein phosphate type 2A. Taking this into account, in vitro inhibition assays for the stable compounds (**1** and **3**) were undertaken. These indicated that while (+)-longilene peroxide (**1**) showed inhibition on the Ser-Thr protein phosphatase type 2A (PP2A) (IC_50_ 11.3 μM ±1.4), (+)-prelongilene (**3**) was inactive at a concentration of 100 μM.

In an attempt to understand the structural features that are underpinning the observed in vitro inhibitory activities, molecular docking simulations were performed to obtain the most favourable binding positions of the two ligands, (+)-longilene peroxide (**1**) and (+)-prelongilene (**3**), which are inside the Ser-Thr protein phosphatase type 2A (PP2A) binding-pocket enzyme. It has to be noted that PP2A is a metal-dependent enzyme that includes two Mn^2+^ ions within its active site. Metalloproteins are still challenging systems that can be tackled through molecular simulations, mainly because the atomic charges of those atoms that were included within the metal coordination sphere are difficult to parametrize. For that reason, electrostatic charges were established for those residues involved in a coordination sphere within 3 Å from the metal ion (Asp-57, His-59, Asp-85, Asn-117, His-167, His-241 and H_2_O-503), together with those of the two Mn^2+^ ions by ab initio calculations at the B3LYP/6-31G** theoretical level. Next, each inhibitor was docked into the active site of PP2A using the AutoDock software [15,16]. The structures that were resulting from the docking simulations were clustered on the basis of their binding energies and geometrical similarity. Nevertheless, all of the docking solutions where the ligand was located less than 4.5 Å from the Mn^2+^ ions were discarded. The criterion for this was that examination of different crystallographic structures showed that the distance between the ligand and the manganese ions was larger than 4.5 Å in all of them. Finally, in order to identify major contributions to binding, selected docking scenarios were evaluated using DrugScore [17]. As a control for the docking simulations, the structure of the complexes formed by okadaic acid—PP2A (pdb 2ie4) and calyculin—PP2A (pdb 1IT6) was predicted using the previous protocol, obtaining excellent results through comparing the corresponding crystallographic coordinates (RMSD 0.47 and 0.5 Å, respectively) (see Supplementary Materials) [18,19].

**Figure 4:**
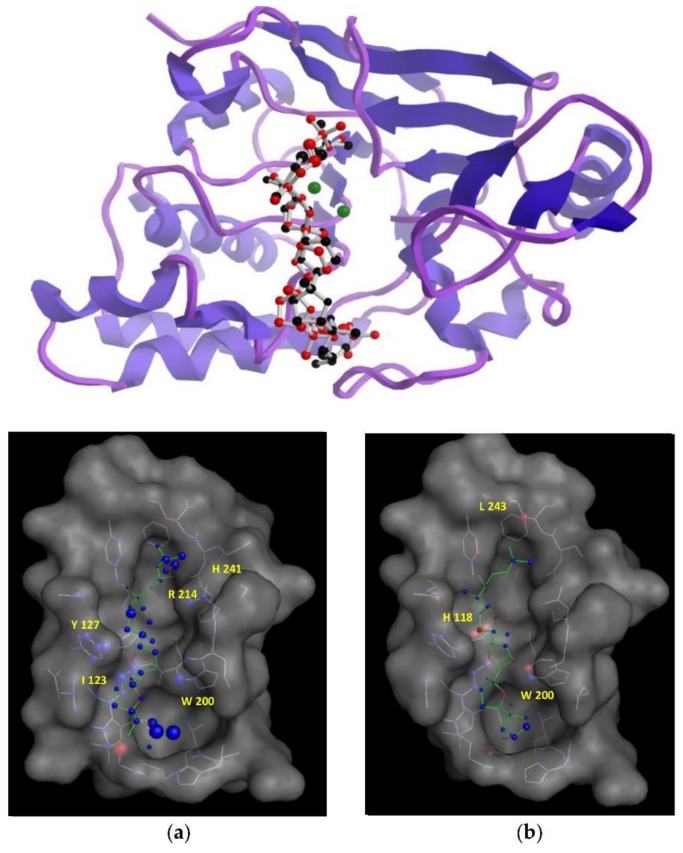
*(Top)* Structures minimum energy obtained of the molecular docking of the PP2A with the (+)-longilene peroxide (**1**, red) and the (+)-prelongilene (**3**, black). (*Bottom*) Analysis in *DrugScore* of the structures minimum energy obtained of the molecular *Docking* of the PP2A with the (+)-longilene peroxide (**1**) (**a**), and the (+)-prelongilene (**3**) (**b**).

According to our simulation results (Figure 4), the binding modes of **1** and **3** to PP2A are very similar, as expected from their minor structural differences. However, the resulting (+)-longilene peroxide (**1**)—PP2A complex showed several favourable contacts that were not observed in the (+)-prelongilene (**3**)—PP2A complex. Among these, were the key hydrogen bonds that were formed between the hydroperoxide group with the Arg-214 and His-241 residues, as well as that between the hydroxyl group on C-23 and the Ile-123 residue. On the other hand, from the analysis of the structure of the (+)-prelongilene—PP2A complex, no important favourable contributions were identified, but small unfavourable contacts with Trp-200, Hys-118 and Leu-243 were observed. The overall orientation of the two ligands within the active site of PP2A was therefore very similar and the structural difference between these two molecules is the additional allylic hydroperoxide group at C-2 in **1**. For these reasons, it seems clear that the two predicted hydrogen bonds in the (+)-longilene peroxide (**1**)—PP2A complex are the factor leading to their differences in bioactivity.

## 3. Materials and Methods

### 3.1. General Experimental Procedures

Optical rotations were determined on a Perkin-Elmer 241 polarimeter (Waltham, MA, USA), using a sodium lamp operating at 589 nm. The IR spectrum was measured on a Bruker IFS55 spectrometer (Billerica, MA, USA), using a chloroform solution to place a film of the compounds on the NaCl disk. UV spectra were determined on a spectrophotometer V-560, Jasco Inc. (Easton, MD, USA) NMR spectra were obtained on Bruker AVANCE 500 MHz instruments at 300 K, and coupling constants are given in Hz. NMR experiments, COSY, HSQC, HMBC, and ROESY, were performed using standard pulse sequences. Phase-sensitive ROESY spectra were measured using a mixing time of 500 ms. ^3^*J*_H,H_ values were measured from one-dimensional (1D) ^1^H-NMR. NMR data were processed using Topspin or MestReNova software (v.10., Santiago de Compostela, Spain). Mass spectra were recorded on a VG AutoSpec FISON spectrometer (Danvers, MA, USA). HPLC (High performance liquid chromatography) separations were carried out with a LKB 2248 system (LKB-Producter AB, Bromma, Sweden) that was equipped with a photodiode array detector. All of the solvents used were HPLC-grade. HPLC chromatography was monitored by TLC, performed on AL Si gel Merck 60 F254 (Kenilworth, NJ, USA). TLC plates were visualized by UV light (365 nm) and phosphomolybdic acid solution 10 wt % in methanol.

### 3.2. Biological Material

Specimens of *Laurencia viridis* were collected in April 2016 in the intertidal zone at Paraiso Floral, Tenerife, Canary Islands (28°07′12″ N, 16°46′45″ W). Dried material from the sterile plants, sporophytes, and gametophytes was filed at TFC Phyc 7180. (Herbario de la Universidad de La Laguna, Departamento de Biología Vegetal, Botánica, Tenerife, Spain).

### 3.3. Extraction and Isolation

The dried red algae was extracted with CHCl_3_:MeOH (1:1) at room temperature (rt), and a dark-green, viscous oil was obtained (78.0 g) after concentration under reduced pressure. The extract was first chromatographed using Sephadex LH20 (7 Ø × 50 cm) using DCM/MeOH (1:1) as the mobile phase. This procedure was repeated until all of the extract was processed. The enriched polyether fractions (52.1 g) were collected between 225 and 360 mL was subsequently processed on a silica gel 0.2–0.5 mm (Sigma-Aldrich, St. Louis, MO, USA) column (7 Ø × 50 cm) using a linear gradient of *n*-Hex/EtOAc (8:2–2:8), and the fraction collected between 350 and 500 mL was dried (1.49 g). Next, medium-pressure chromatography was done using Lobar LiChroprep-RP18 MeOH/H_2_O (9:1) at 5 mL/min flow. Fractions collected between 138 and 146 mL, and 147 and 165 mL were pooled together (**5D** and **5E** 100 and 1220 mg, respectively). Final purification was performed on an HPLC with a μ-Porasil column, *n*-Hex/Acetone (8:2) and DCM/Acetone (8:2), to afford (+)-prelongilene (**3**) (2.1 mg) and the compound **5** (0.91 mg), (in the case of the fraction **5D**); while for the fraction **5E**, *n*-Hex/EtOAc (7:3) and DCM/Acetone (8:2) were used to afford the polyethers: (+)-longilene peroxide (**1**) (3.1 mg), longilene (**2**) (1.5 mg), and the compound **4** (0.83 mg) (see Supplementary Materials).

*(+)-Longilene peroxide* (**1**): Amorphous white solid, ${\left[\alpha \right]}_{D}^{25}$ +47 (*c* 0.31, CHCl_3_); IR *v*_max_ (CHCl_3_) 3360, 2969, 1732, 1455, 1373 and 1077 cm^−1^; HR-ESI-MS *m/z* 563.3571 [M + Na]^+^ (calcd. 563.3559 for C_30_H_52_O_8_Na); NMR data ^1^H (500 MHz, CDCl_3_) and ^13^C (125 MHz, CDCl_3_) see Table 1.

*Longilene* (**2**): Amorphous white solid; ${\left[\alpha \right]}_{D}^{25}$ +21 (*c* 0.15, CHCl_3_); IR *v*_max_ (CHCl_3_) 3365, 2971, 2929, 2872, 1455, 1373 and 1085 cm^−1^; HR-ESI-MS *m/z* 524.3716 [M]^+^ (calcd. 524.3713 for C_30_H_52_O_7_); NMR data ^1^H (500 MHz, CDCl_3_) and ^13^C (125 MHz, CDCl_3_) see Table 1.

*(+)-Prelongilene* (**3**): Amorphous white solid, ${\left[\alpha \right]}_{D}^{25}$ +19 (*c* 0.21, CHCl_3_); IR *v*_max_ (CHCl_3_) 3363, 2971, 2931, 2872, 1453, 1372 and 1082 cm^−1^; HR-ESI-MS *m/z* 531.3655 [M + Na]^+^ (calcd. 531.3662 for C_30_H_52_O_6_Na); NMR data ^1^H (500 MHz, CDCl_3_) and ^13^C (125 MHz, CDCl_3_) see Table 2.

### 3.4. Transformation of (+)-Prelongilene (***3***) into Compound ***5***

Dowex-50 was added to a solution of **3** (1.1 mg, 2.2 μmol) in CDCl_3_ (0.4 mL), and the contents were mixed for 48 h at room temperature, while monitoring the reaction by NMR. Of the resulting solution was 0.91 mg (1.9 μmol) of pure compound **5** (see Supplementary Materials).

### 3.5. Protein Phosphatase 2A Inhibition Assay

The enzymatic assays were performed in 96-well plates. Each well contained 10 μL of MnCl_2_ (1 mM), 5 μL of BSA (5 mg/mL), 10 μL of buffer (50 mM Tris-HCl, 0.1 mM EDTA, 5 mM DTT, 0.025% Tween, pH 7.0), 15 μL of distilled water, and 5 μL of PP2A (Sigma-Aldrich^®^; 50 mM of Tris-HCl pH 7.0, 14 mM of 2-mercaptoethanol, 1 mM of benzamidine, 0.1 mM of PMSF, 1 mM of EDTA y 50% of glycerol). The blank was prepared in the same way as the sample, but with no enzyme (30 μL of distilled water); there were at least eight blanks per plate. The reaction mixture was incubated for 10 min at 37 °C, sample (10 μL) was added, except to the control and blank wells. This mixture was again incubated for 10 min at 37 °C and then the fluorescent substrate (PNPP) was added in 50 μL as an assay buffer at a final concentration of 1.5 mg/mL. Each determination was carried out in triplicate and each sample was tested in four different dilutions (100, 10, 1, and 0.1 µM). The reaction was incubated for 60 min at 37 °C and fluorescence was measured using a microplate reader fluorometer set at 492 nm (exc) and 514 nm (em).

### 3.6. Docking Studies 

Docking simulations were performed with Autodock software (v.3.0.5; Molecular Graphics Laboratory, The Scripps Research Institute, La Jolla, CA, USA) using the GUI AutoDockTools (v.1.5.2). The receptor file was prepared using the coordinates of the crystallographic structure OA-PP2A (pdb 2ie4) that was obtained from the Protein Data Bank in which the ligand was removed. The atomic charges of the Mn^2+^ ions as well as that of those residues in their coordination spheres were calculated by ab initio calculations at the B3LYP/6-31G** theoretical level.

## 4. Conclusions

Three new squalene derived metabolites have been isolated from the red algae *Laurencia. viridis* and their structures were determined on the basis of a spectroscopic analysis. Subsequently, the protein phosphatase type 2A inhibitory activity of the less labile compounds (**1** and **3**) was tested, resulting in **1** as a moderately active compound (IC_50_ 11.3 μM), while the very similar compound **3** was completely inactive up to a concentration of 100 μM. Due to the minor structural changes between them, it is clear that the allylic hydroperoxide functional group of **1** is responsible for the observed differences in activity.

## Figures and Tables

**Table 1:** NMR data for (+)-longilene peroxide (**1**) and longilene (**2**) (500 MHz, 125 MHz, CDCl_3_).

	(+)-Longilene Peroxide (1)		Longilene (2)	
Position	δ_C_, Type	δ_H_ (*J* in Hz)	δ_C_, Type	δ_H_ (*J* in Hz)
**1**	26.9, CH_3_	1.19, s	29.6, CH_3_	1.29, s
**2**	80.1, C		70.4, C	
**3**	137.0, CH	5.43, d (15.6)	141.2, CH	5.62, d (15.6)
**4**	125.8, CH	5.81, ddd (6.6, 8.5, 15.6)	122.0, CH	5.77, ddd (7.0, 7.4, 15.6)
**5**	41.3, CH_2_	1.78, dd (8.5, 13.3) 2.20, dd (6.6, 13.3)	40.4, CH_2_	1.78, dd (7.0, 13.4) 2.15, dd (7.4, 13.4)
**6**	73.9, C		74.0, C	
**7**	85.1, CH	3.72, m	84.9, CH	3.70, dd (6.4, 6.6)
**8**	25.8, CH_2_	1.89, m 2.06, m	25.6, CH_2_	1.91, m 2.03, m
**9**	29.7, CH_2_	1.49, m 2.06, m	30.0, CH_2_	1.47, m 2.06, m
**10**	85.8, C		85.7, C	
**11**	85.8, CH	4.09, m	85.4, CH	4.11, dd (5.5, 5.6)
**12**	30.1, CH_2_	1.50, m 2.01, m	30.0, CH_2_	1.49, m 2.01, m
**13**	29.9, CH_2_	1.50, m 2.01, m	30.0, CH_2_	1.49, m 2.01, m
**14**	85.4, CH	4.09, m	85.4, CH	4.11, dd (5.5, 5.6)
**15**	85.4, C		85.7, C	
**16**	29.3, CH_2_	1.46, m 2.03, m	30.0, CH_2_	1.47, m 2.06, m
**17**	25.2, CH_2_	1.89, m 2.03, m	25.6, CH_2_	1.91, m 2.03, m
**18**	84.1, CH	3.72, m	84.9, CH	3.70, dd (6.4, 6.6)
**19**	73.8, C		74.0, C	
**20**	40.8, CH_2_	1.88, dd (6.8, 13.4) 2.20, dd (7.0, 13.4)	40.4, CH_2_	1.78, dd (7.0, 13.4) 2.15, dd (7.4, 13.4)
**21**	122.2, CH	5.75, ddd (6.8, 7.0, 15.6)	122.2, CH	5.77, ddd (7.0, 7.4, 15.6)
**22**	141.2, CH	5.61, d (15.6)	141.2, CH	5.62, d (15.6)
**23**	70.0, C		70.4, C	
**24**	29.4, CH_3_	1.27, s	29.6, CH_3_	1.29, s
**25**	24.2, CH_3_	1.37, s	29.8, CH_3_	1.31, s
**26**	24.3, CH_3_	1.20, s	24.1, CH_3_	1.24, s
**27**	24.2, CH_3_	1.09, s	23.7, CH_3_	1.10, s
**28**	23.6, CH_3_	1.07, s	23.7, CH_3_	1.10, s
**29**	24.3, CH_3_	1.27, s	24.1, CH_3_	1.24, s
**30**	29.6, CH_3_	1.31, s	29.8, CH_3_	1.31, s
**-OOH**		10.57, s		
**-OH-6**		5.24, s		
**-OH-19**		5.03, s		
**-OH-23**		3.29, s		

**Table 2:** NMR data for (+)-prelongilene (**3**) (500 MHz, 125 MHz, CDCl_3_).

	(+)-Prelongilene (3)				
Position	δ_C_ Type	δ_H_ (*J* in Hz)	C	δ_C_ Type	δ_H_ (*J* in Hz)
**1**	17.7, CH_3_	1.60, s	**16**	30.0, CH_2_	1.46, m 2.06, m
**2**	131.1, C		**17**	25.5, CH_2_	1.93, m 2.14, m
**3**	124.8, CH	5.08, t (6.9)	**18**	85.0, CH	3.81, dd (4.4, 8.1)
**4**	22.5, CH_2_	1.97, m 2.04, m	**19**	74.1, C	
**5**	39.0, CH_2_	1.29, m 1.43, m	**20**	40.8, CH_2_	1.83, m 2.17, m
**6**	72.5, C		**21**	122.7, CH	5.74, ddd (6.2, 8.6, 15.2)
**7**	83.4, CH	3.73, dd (6.9, 7.6)	**22**	141.0, CH	5.62, d (15.2)
**8**	25.4, CH_2_	1.80, m 1.91, m	**23**	70.3, C	
**9**	30.0, CH_2_	1.46, m 2.04, m	**24**	29.8, CH_3_	1.31, s
**10**	84.7, C		**25**	25.7, CH_3_	1.66, s
**11**	85.0, CH	4.07, dd (5.4, 10.3)	**26**	24.9, CH_3_	1.27, s
**12**	29.5, CH_2_	1.50, m 1.99, m	**27**	23.5, CH_3_	1.08, s
**13**	29.5, CH_2_	1.50, m 1.99, m	**28**	24.0, CH_3_	1.11, s
**14**	85.7, CH	4.13, dd (2.1, 5.7)	**29**	24.1, CH_3_	1.20, s
**15**	85.8, C		**30**	30.1, CH_3_	1.31, s
			**OH-6**		4.94, s
			**OH-19**		4.39, s
			**OH-23**		2.57, s

## Supplementary Materials

The following are available online at http://www.mdpi.com/1660-3397/16/4/131/s1, Table S1: NMR data of (+)-longilene peroxide (**1**) in CDCl_3_ at 300 K, 500 MHz; Table S2: NMR data of longilene (**2**) in CDCl_3_ at 300 K, 500 MHz; Table S3: NMR data of (+)-prelongilene (**3**) in CDCl_3_ at 300 K, 500 MHz; Table S4: NMR data of compound **5** in CDCl_3_ at 300 K, 500 MHz; Table S5: NMR data of compound **4** in CDCl_3_ at 300 K, 500 MHz; Figure S1: ^1^H-NMR spectrum of (+)-longilene peroxide (**1**) in CDCl_3_ at 300 K, 500 MHz; Figure S2: COSY spectrum of (+)-longilene peroxide (**1**) in CDCl_3_ at 300 K, 500 MHz; Figure S3: HSQC spectrum of (+)-longilene peroxide (**1**) in CDCl_3_ at 300 K, 500 MHz; Figure S4: HMBC spectrum of (+)-longilene peroxide (**1**) in CDCl_3_ at 300 K, 500 MHz; Figure S5: ROESY spectrum of (+)-longilene peroxide (**1**) in CDCl_3_ at 300 K, 500 MHz; Figure S6: MS spectrum of (+)-longilene peroxide (**1**); Figure S7: ^1^H-NMR spectrum of longilene (**2**) in CDCl_3_ at 300 K, 500 MHz; Figure S8: COSY spectrum of longilene (**2**) in CDCl_3_ at 300 K, 500 MHz; Figure S9: HSQC spectrum of longilene (**2**) in CDCl_3_ at 300 K, 500 MHz; Figure S10: HMBC spectrum of longilene (**2**) in CDCl_3_ at 300 K, 500 MHz; Figure S11: MS spectrum of longilene (**2**); Figure S12: ^1^H-NMR spectrum of (+)-prelongilene (**3**) in CDCl_3_ at 300 K, 500 MHz; Figure S13: COSY spectrum of (+)-prelongilene (**3**) in CDCl_3_ at 300 K, 500 MHz; Figure S14: HSQC spectrum of (+)-prelongilene (**3**) in CDCl_3_ at 300 K, 500 MHz; Figure S15: HMBC spectrum of (+)-prelongilene (**3**) in CDCl_3_ at 300 K, 500 MHz; Figure S16: ROESY spectrum of (+)-prelongilene (**3**) in CDCl_3_ at 300 K, 500 MHz; Figure S17: MS spectrum of (+)-prelongilene (**3**); Figure S18: Conversion of (+)-prelongilene (**3**) to compound **5** for ^1^H-NMR; 0 h; Figure S19: Conversion of (+)-prelongilene (**3**) to compound **5** for ^1^H-NMR; 24 h; Figure S20: Conversion of (+)-prelongilene (**3**) to compound **5** for ^1^H-NMR; 48 h; Figure S21: Conversion of (+)-prelongilene (**3**) to compound **5** for ^1^H-NMR; 72 h; Figure S22: ^1^H-NMR spectrum of compound **5** in CDCl_3_ at 300 K, 500 MHz; Figure S23: COSY spectrum of compound **5** in CDCl_3_ at 300 K, 500 MHz; Figure S24: HSQC spectrum of compound **5** in CDCl_3_ at 300 K, 500 MHz; Figure S25: HMBC spectrum of compound **5** in CDCl_3_ at 300 K, 500 MHz; Figure S26: NOESY spectrum of compound **5** in CDCl_3_ at 300 K, 500 MHz; Figure S27: MS spectrum of compound **5**; Figure S28: ^1^H-NMR spectrum of compound **4** in CDCl_3_ at 300 K, 500 MHz; Figure S29: HSQC spectrum of compound **4** in CDCl_3_ at 300 K, 500 MHz; Figure S30: HMBC spectrum of compound **4** in CDCl_3_ at 300 K, 500 MHz; Figure S31: NOESY spectrum of compound **4** in CDCl_3_ at 300 K, 500 MHz; Figure S32: In vitro inhibitory effect of (+)-longilene peroxide (**1**) on PP2A.
