# Supplementary material for: Marine Longilenes, Oxasqualenoids with Ser-Thr Protein Phosphatase 2A Inhibition Activity
Source: Mar Drugs. 2018 Apr 17;16(4):131. doi: 10.3390/md16040131 (PMC5923418; doi:10.3390/md16040131)

Supporting Information

Marine Longilenes, Oxasqualenoids with PP2A Inhibition Activity

Francisco Cen-Pacheco[1,2*], Claudia P. Manríquez[1,3], María L. Souto[1,4], Manuel Norte[1,4], José J. Fernández[1,4*], Antonio Hernández Daranas[1,5*]

1 Instituto Universitario de Bio-Orgánica Antonio González (IUBO AG), Centro de Investigaciones Biomédicas de Canarias (CIBICAN), Universidad de La Laguna (ULL), Avenida Astrofísico Francisco Sánchez 2, 38206 Tenerife, Spain; msouto@ull.edu.es (M.L.S.); mnorte@ull.edu.es (M.N.)

2 Facultad de Bioanálisis, Campus-Veracruz, Universidad Veracruzana, 91700 Veracruz, Mexico; fcen@uv.mx (F.C.P.)

3 Facultad de Ciencias Naturales y Oceanográficas, Departamento de Botánica, Universidad de Concepción, Barrio Universitario, Concepción, Región del Biobío, Chile; claudiaperez@udec.cl (C.P.M.)

4 Departamento de Química Orgánica, Universidad de La Laguna (ULL), Avenida Astrofísico Francisco Sánchez s/n, 38206 Tenerife, Spain

5 Instituto de Productos Naturales y Agrobiología (IPNA), CSIC, Avenida Astrofísico Francisco Sánchez 2, 38206 Tenerife, Spain

* Correspondence: jjfercas@ull.edu.es (J.J.F.); adaranas@ipna.csic.es (A.H.D.); Tel.: +34-922-318-586

INDEX	
**Table S1**. NMR data of (+)-longilene peroxide (**1**) in $CDCl_3$ at 300 K, 500 MHz.	3
**Figure S1**. ^{1}H-NMR spectrum of (+)-longilene peroxide (**1**) in $CDCl_3$ at 300 K, 500 MHz.	4
**Figure S2**. COSY spectrum of (+)-longilene peroxide (**1**) in $CDCl_3$ at 300 K, 500 MHz.	5
**Figure S3**. HSQC spectrum of (+)-longilene peroxide (**1**) in $CDCl_3$ at 300 K, 500 MHz.	6
**Figure S4**. HMBC spectrum of (+)-longilene peroxide (**1**) in $CDCl_3$ at 300 K, 500 MHz.	7
**Figure S5**. ROESY spectrum of (+)-longilene peroxide (**1**) in $CDCl_3$ at 300 K, 500 MHz.	8
**Figure S6**. MS spectrum of (+)-longilene peroxide (**1**).	9
**Table S2**. NMR data of longilene (**2**) in $CDCl_3$ at 300 K, 500 MHz.	10
**Figure S7**. ^{1}H-NMR spectrum of longilene (**2**) in $CDCl_3$ at 300 K, 500 MHz.	11
**Figure S8**. COSY spectrum of longilene (**2**) in $CDCl_3$ at 300 K, 500 MHz.	12
**Figure S9**. HSQC spectrum of longilene (**2**) in $CDCl_3$ at 300 K, 500 MHz.	13
**Figure S10**. HMBC spectrum of longilene (**2**) in $CDCl_3$ at 300 K, 500 MHz.	14
**Figure S11**. MS spectrum of longilene (**2**).	15
**Table S3**. NMR data of (+)-prelongilene (**3**) in $CDCl_3$ at 300 K, 500 MHz.	16
**Figure S12**. ^{1}H-NMR spectrum of (+)-prelongilene (**3**) in $CDCl_3$ at 300 K, 500 MHz.	17
**Figure S13**. COSY spectrum of (+)-prelongilene (**3**) in $CDCl_3$ at 300 K, 500 MHz.	18

**Figure S14**. HSQC spectrum of (+)-prelongilene (**3**) in $CDCl_3$ at 300 K, 500 MHz.	**19**
**Figure S15**. HMBC spectrum of (+)-prelongilene (**3**) in $CDCl_3$ at 300 K, 500 MHz.	**20**
**Figure S16**. ROESY spectrum of (+)-prelongilene (**3**) in $CDCl_3$ at 300 K, 500 MHz.	**21**
**Figure S17**. MS spectrum of (+)-prelongilene (**3**).	**22**
**Figure S18**. Conversion of (+)-prelongilene (**3**) to compound **5** for ^1H NMR; 0 h.	**23**
**Figure S19**. Conversion of (+)-prelongilene (**3**) to compound **5** for ^1H NMR; 24 h.	**23**
**Figure S20**. Conversion of (+)-prelongilene (**3**) to compound **5** for ^1H NMR; 48 h.	**24**
**Figure S21**. Conversion of (+)-prelongilene (**3**) to compound **5** for ^1H NMR; 72 h.	**24**
**Table S4**. NMR data of compound **5** in $CDCl_3$ at 300 K, 500 MHz.	**25**
**Figure S22**. ^1H-NMR spectrum of compound **5** in $CDCl_3$ at 300 K, 500 MHz.	**26**
**Figure S23**. COSY spectrum of compound **5** in $CDCl_3$ at 300 K, 500 MHz.	**27**
**Figure S24**. HSQC spectrum of compound **5** in $CDCl_3$ at 300 K, 500 MHz.	**28**
**Figure S25**. HMBC spectrum of compound **5** in $CDCl_3$ at 300 K, 500 MHz.	**29**
**Figure S26**. NOESY spectrum of compound **5** in $CDCl_3$ at 300 K, 500 MHz.	**30**
**Figure S27**. MS spectrum of compound **5**.	**31**
**Table S5**. NMR data of compound **4** in $CDCl_3$ at 300 K, 500 MHz.	**32**
**Figure S28**. ^1H-NMR spectrum of compound **4** in $CDCl_3$ at 300 K, 500 MHz.	**33**
**Figure S29**. HSQC spectrum of compound **4** in $CDCl_3$ at 300 K, 500 MHz.	**34**
**Figure S30**. HMBC spectrum of compound **4** in $CDCl_3$ at 300 K, 500 MHz.	**35**
**Figure S31**. NOESY spectrum of compound **4** in $CDCl_3$ at 300 K, 500 MHz.	**36**
**Figure S32.** *In vitro* inhibitory effect of (+)-longilene peroxide (**1**) on PP2A	**37**

Table S1. NMR data of (+)-longilene peroxide (**1**) in $CDCl_3$ at 300 K, 500 MHz.

Carbon	δ ^{13}C	Multiplicity	δ ^{1}H	*J* in Hz
**1**	26.9	CH_3	1.19	s
**2**	80.1	C		
**3**	137.0	CH	5.43	d 15.6
**4**	125.8	CH	5.81	ddd 6.6, 8.5, 15.6
**5**	41.3	CH_2	1.78<br>2.20	dd 8.5, 13.3<br>dd 6.6, 13.3
**6**	73.9	C		
**7**	85.1	CH	3.72	m
**8**	25.8	CH_2	1.89<br>2.06	m<br>m
**9**	29.7	CH_2	1.49<br>2.06	m<br>m
**10**	85.8	C		
**11**	85.8	CH	4.09	m
**12**	30.1	CH_2	1.50<br>2.01	m<br>m
**13**	29.9	CH_2	1.50<br>2.01	m<br>m
**14**	85.4	CH	4.09	m
**15**	85.4	C		
**16**	29.3	CH_2	1.46<br>2.03	m<br>m
**17**	25.2	CH_2	1.89<br>2.03	m<br>m
**18**	84.1	CH	3.72	m
**19**	73.8	C		
**20**	40.8	CH_2	1.88<br>2.20	dd 6.8, 13.4<br>dd 7.0, 13.4
**21**	122.2	CH	5.75	ddd 6.8, 7.0, 15.6
**22**	141.2	CH	5.61	d 15.6
**23**	70.0	C		
**24**	29.4	CH_3	1.27	s
**25**	24.2	CH_3	1.37	s
**26**	24.3	CH_3	1.20	s
**27**	24.2	CH_3	1.09	s
**28**	23.6	CH_3	1.07	s
**29**	24.3	CH_3	1.27	s
**30**	29.6	CH_3	1.31	s
**-OOH**			10.57	s
**-OH-6**			5.24	s
**-OH-19**			5.03	s
**-OH-23**			3.29	s

Figure S1. ^{1}H-NMR spectrum of (+)-longilene peroxide (**1**) in $CDCl_3$ at 300 K, 500 MHz.

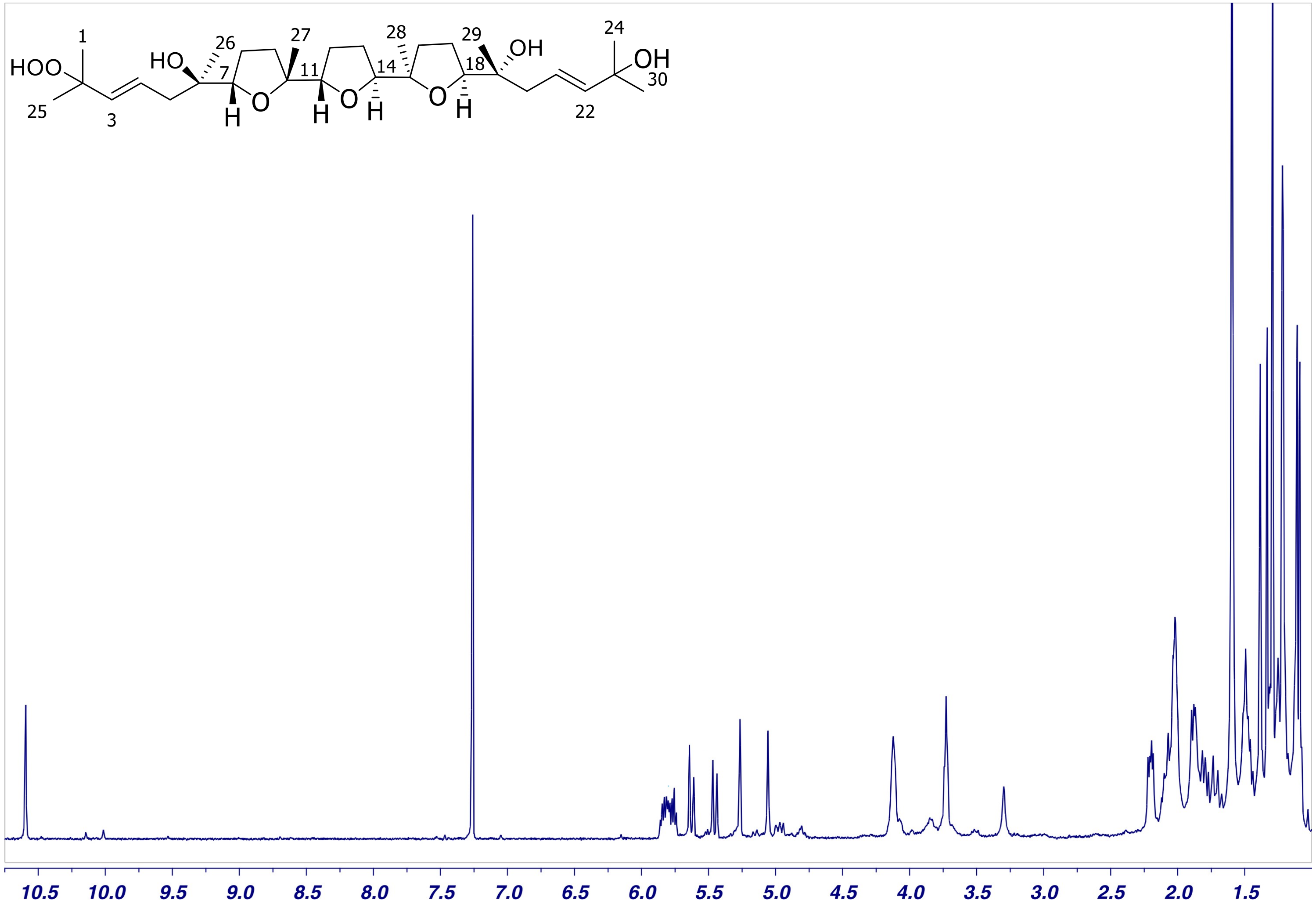

Figure S2. COSY spectrum of (+)-longilene peroxide (**1**) in $CDCl_3$ at 300 K, 500 MHz.

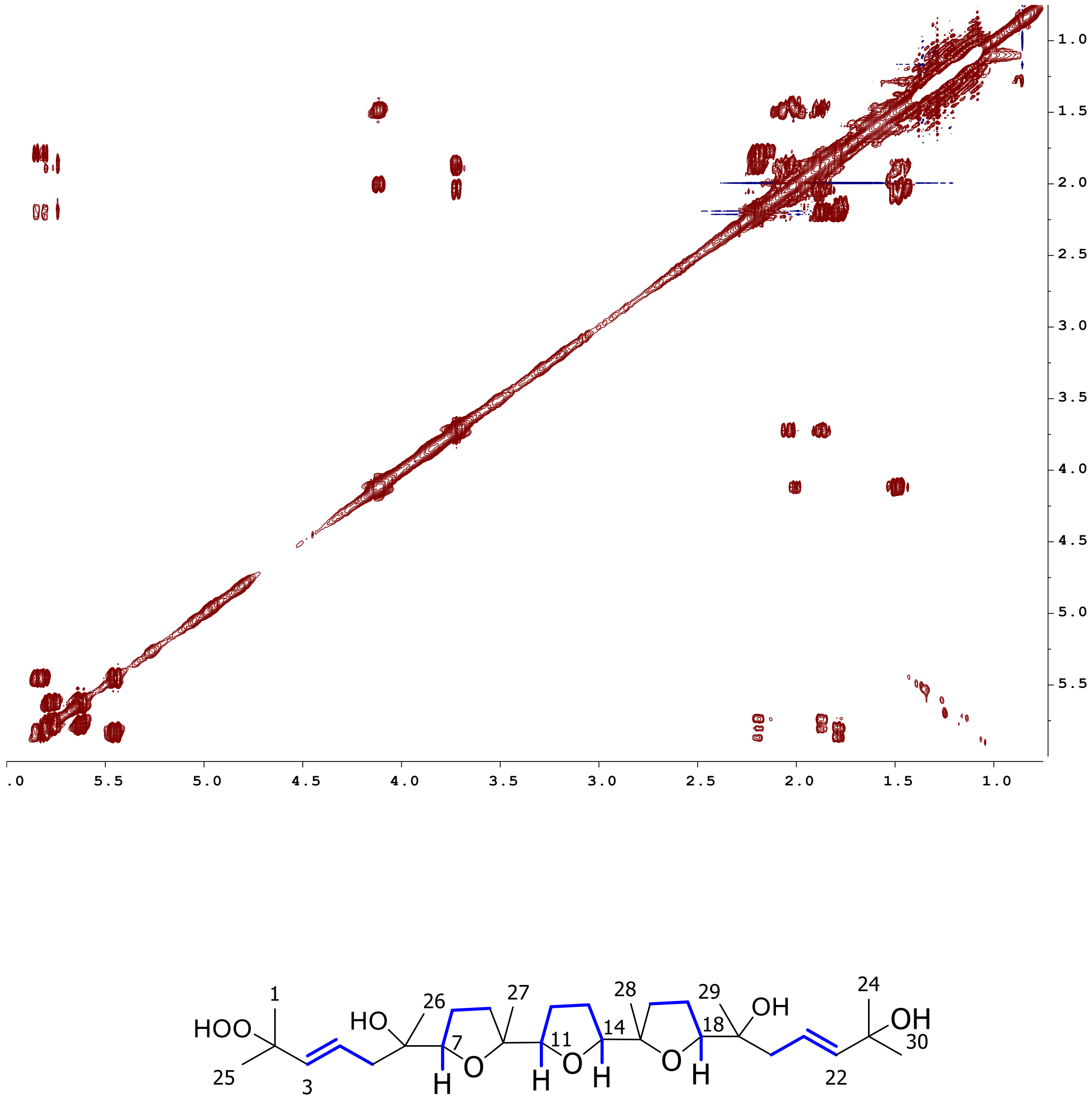

Figure S3. HSQCed spectrum of (+)-longilene peroxide (**1**) in $CDCl_3$ at 300 K, 500 MHz.

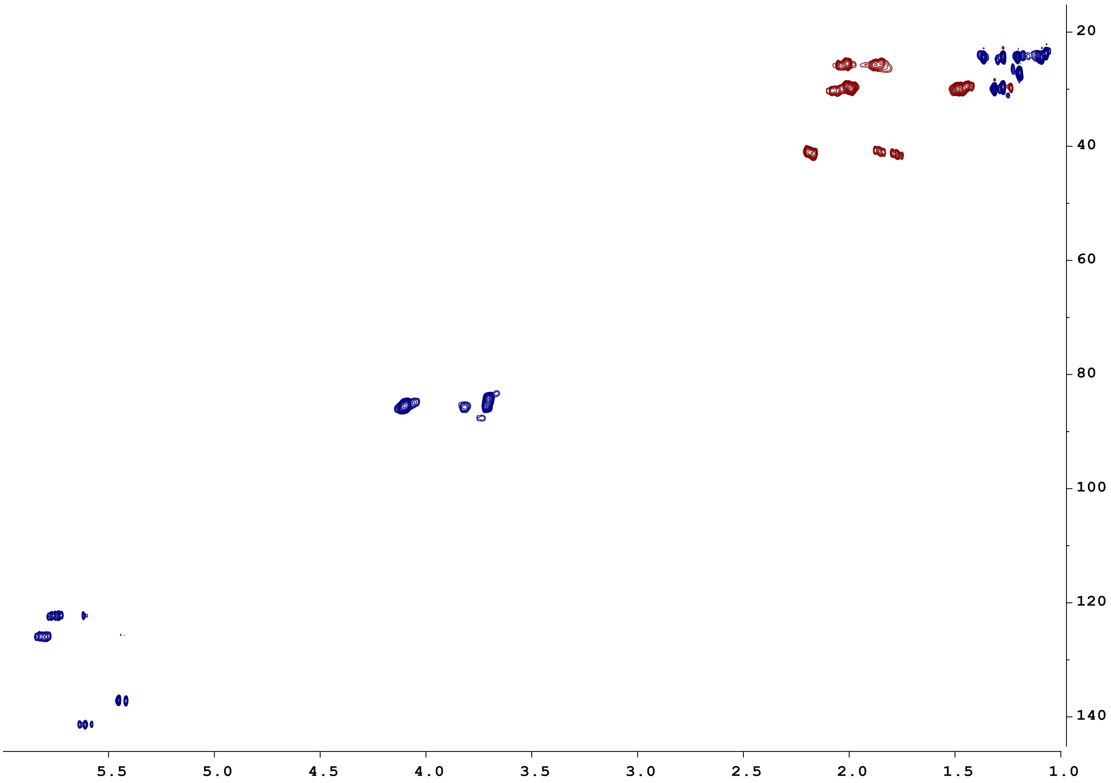

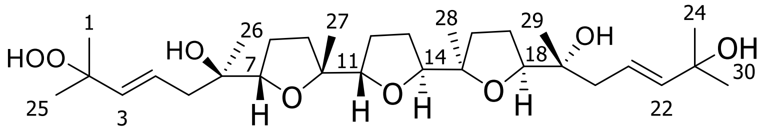

Figure S4. HMBC spectrum of (+)-longilene peroxide (**1**) in $CDCl_3$ at 300 K, 500 MHz.

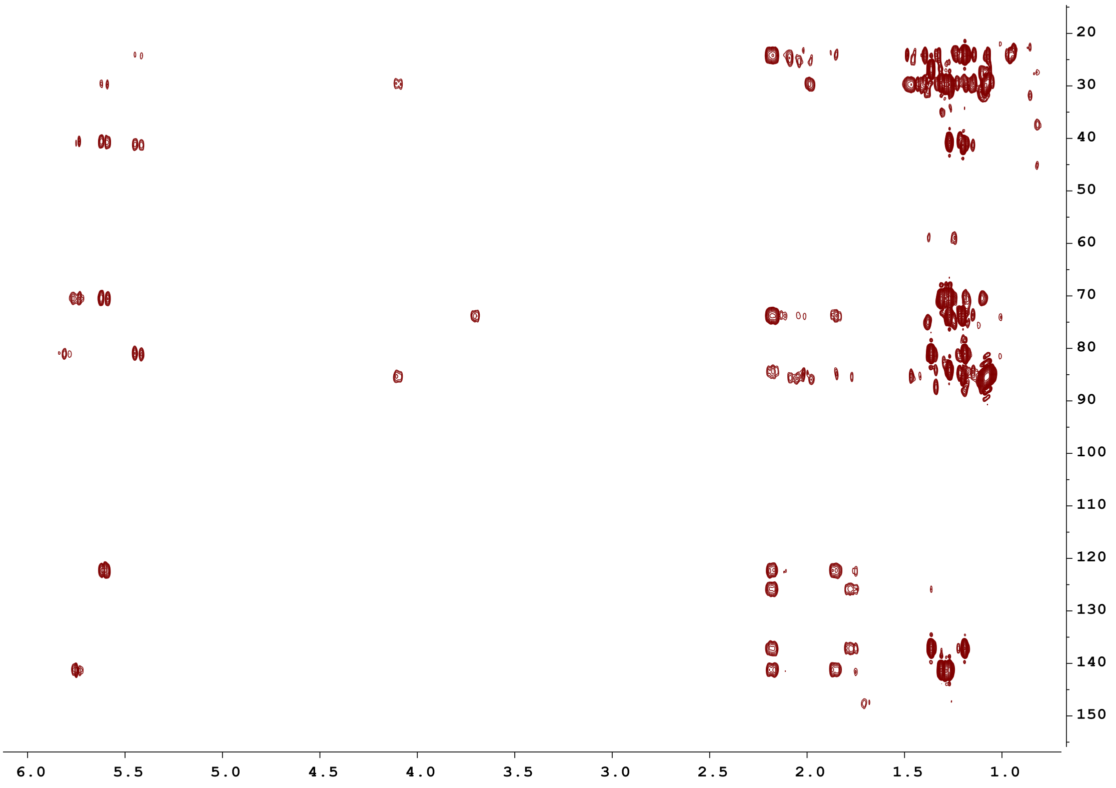

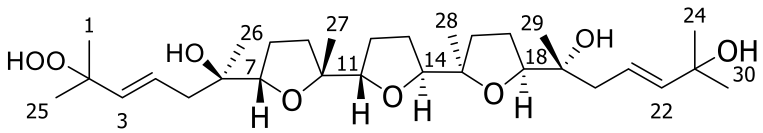

Figure S5. ROESY spectrum of (+)-longilene peroxide (**1**) in $CDCl_3$ at 300 K, 500 MHz.

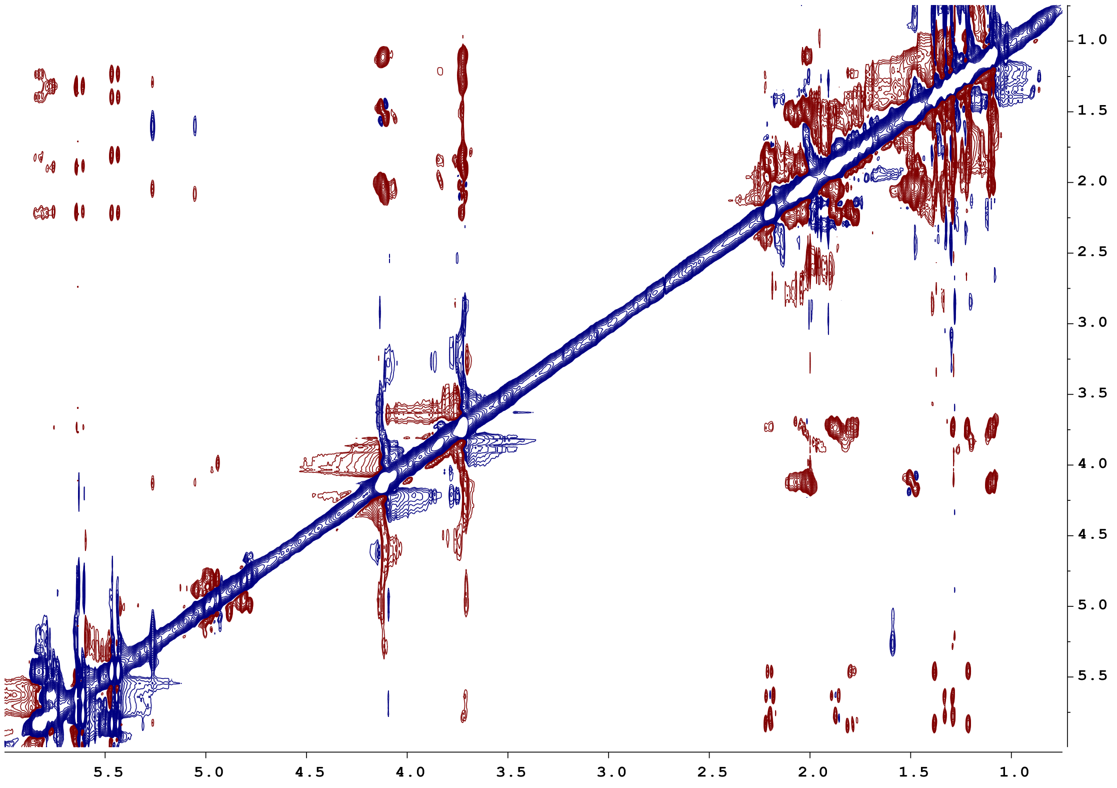

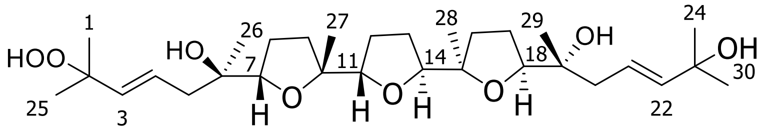

Figure S6. MS spectrum of (+)-longilene peroxide (**1**).

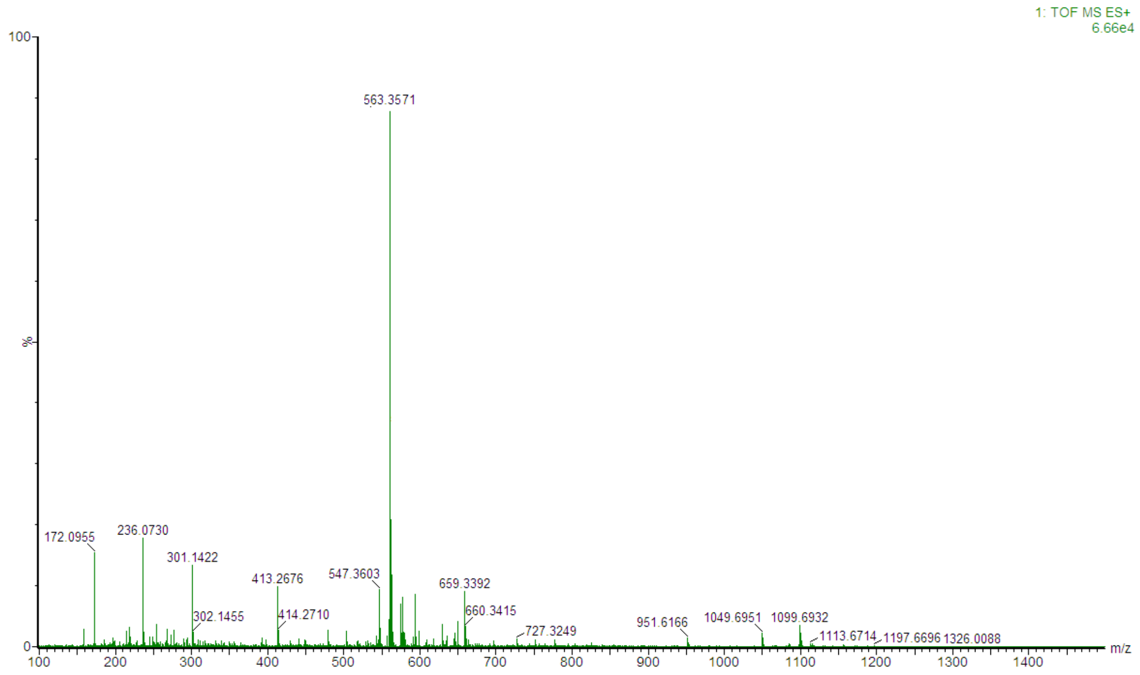

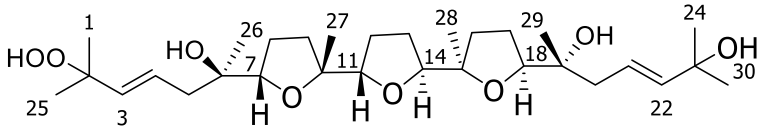

Table S2. NMR data of longilene (**2**) in $CDCl_3$ at 300 K, 500 MHz.

Carbon		δ ^{13}C	Multiplicity	δ ^{1}H	*J* in Hz
**1**	**24**	29.6	CH_3	1.29	s
**2**	**23**	70.4	C		
**3**	**22**	141.2	CH	5.62	d 15.6
**4**	**21**	122.0	CH	5.77	ddd 7.0, 7.4, 15.6
**5**	**20**	40.4	CH_2	1.78<br>2.15	dd 7.0, 13.4<br>dd 7.4, 13.4
**6**	**19**	74.0	C		
**7**	**18**	84.9	CH	3.70	dd 6.4, 6.6
**8**	**17**	25.6	CH_2	1.91<br>2.03	
**9**	**16**	30.0	CH_2	1.47<br>2.06	
**10**	**15**	85.7	C		
**11**	**14**	85.4	CH	4.11	dd 5.5, 5.6
**12**	**13**	30.0	CH_2	1.49<br>2.01	
**25**	**30**	29.8	CH_3	1.31	s
**26**	**29**	24.1	CH_3	1.24	s
**27**	**28**	23.7	CH_3	1.10	s

Figure S7. ^{1}H-NMR spectrum of longilene (**2**) in $CDCl_3$ at 300 K, 500 MHz.

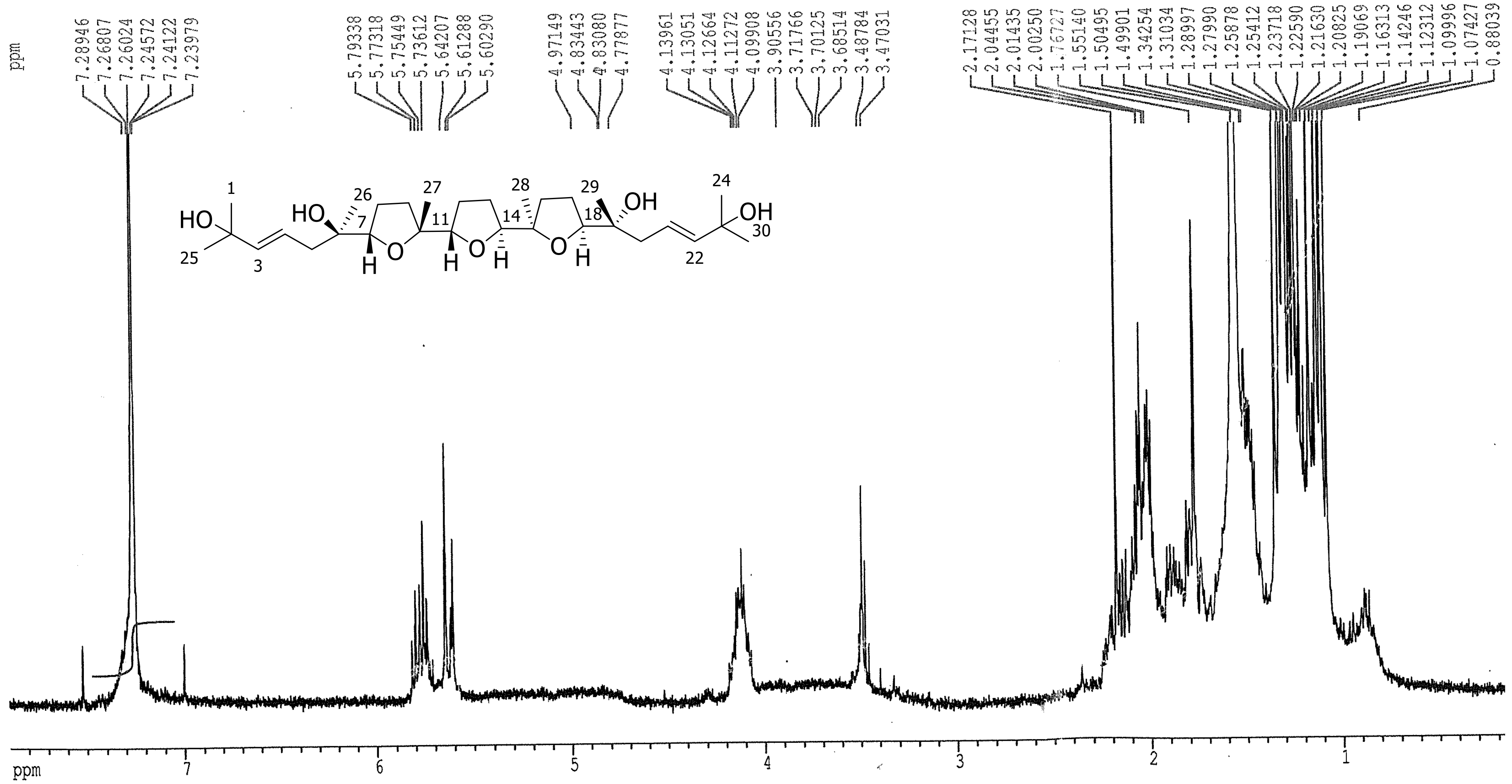

Figure S8. COSY spectrum of longilene (**2**) in $CDCl_3$ at 300 K, 500 MHz.

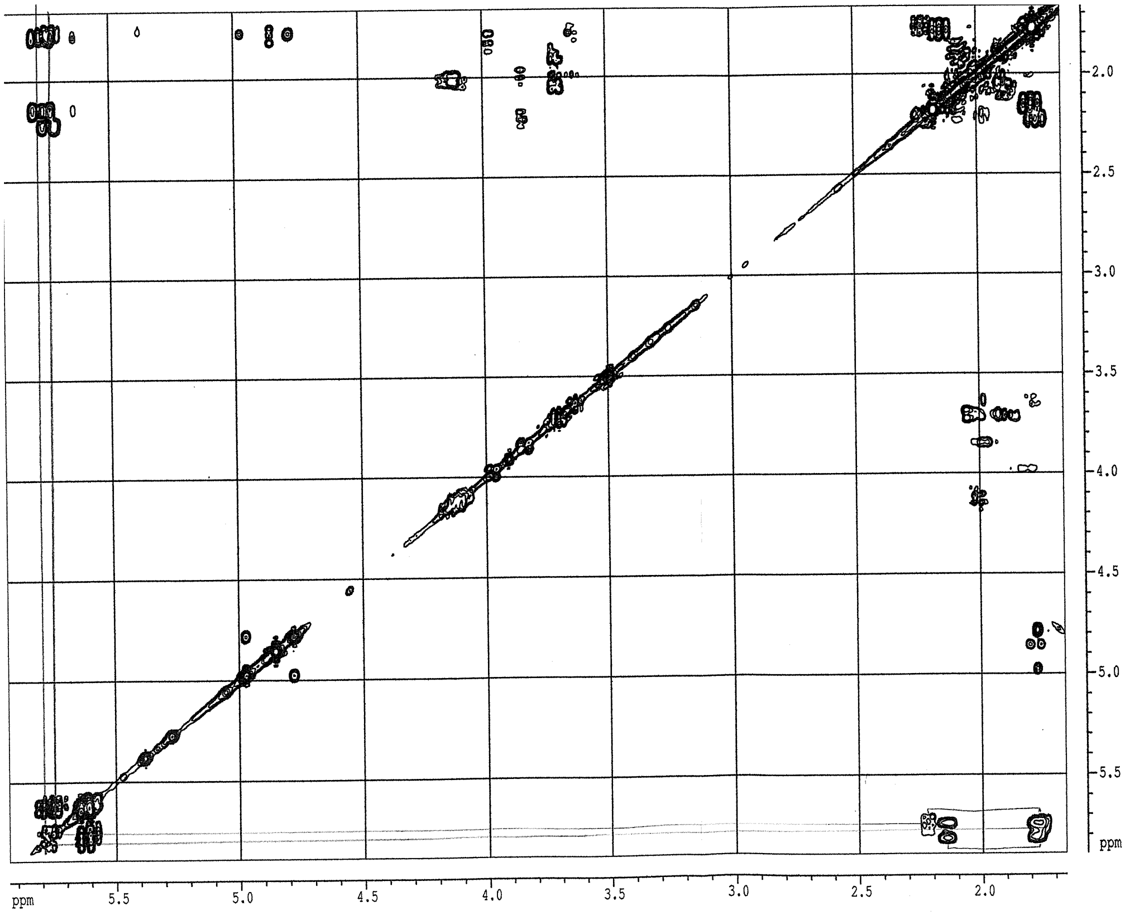

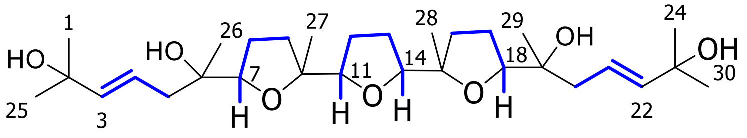

Figure S9. HSQC spectrum of longilene (**2**) in $CDCl_3$ at 300 K, 500 MHz.

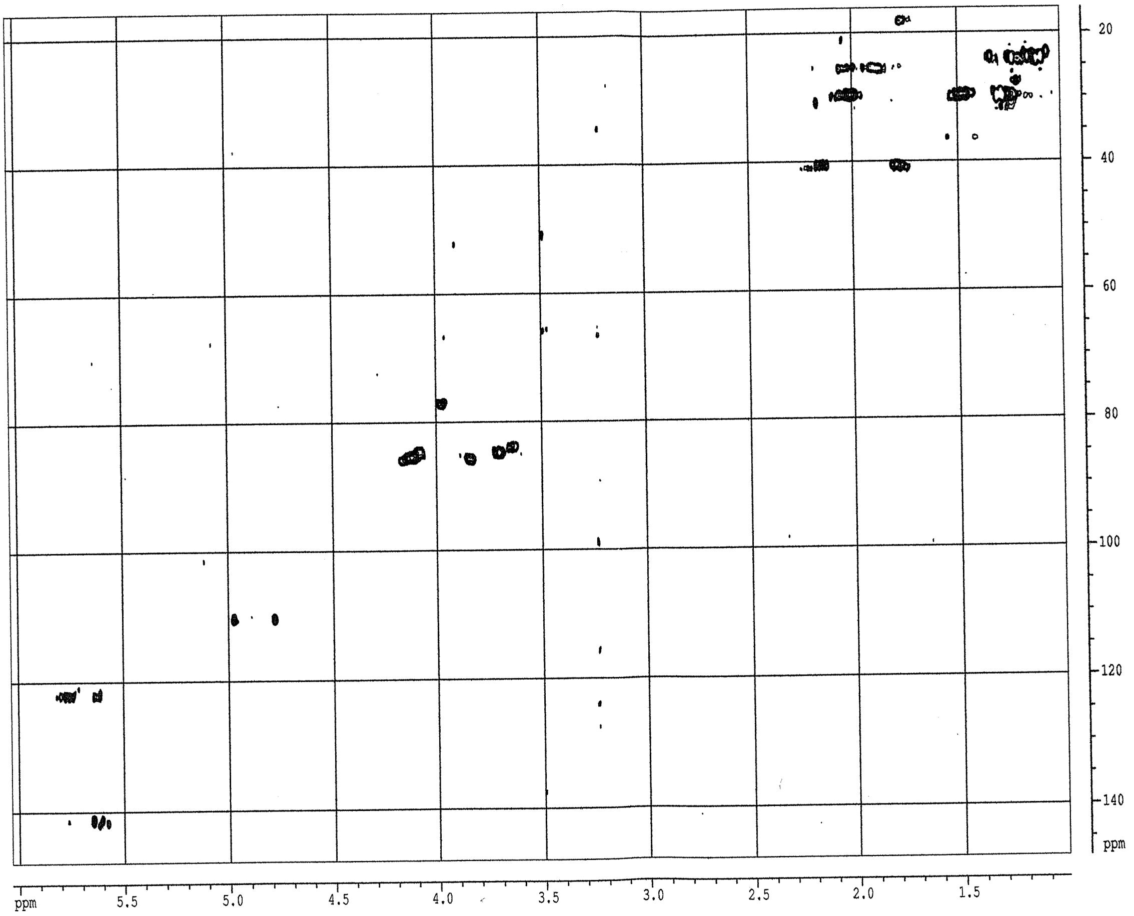

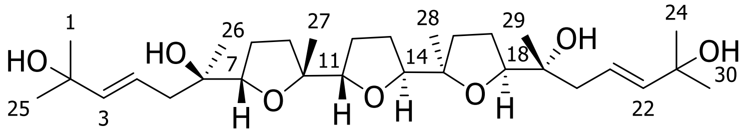

Figure S10. HMBC spectrum of longilene (**3**) in $CDCl_3$ at 298 K, 500 MHz.

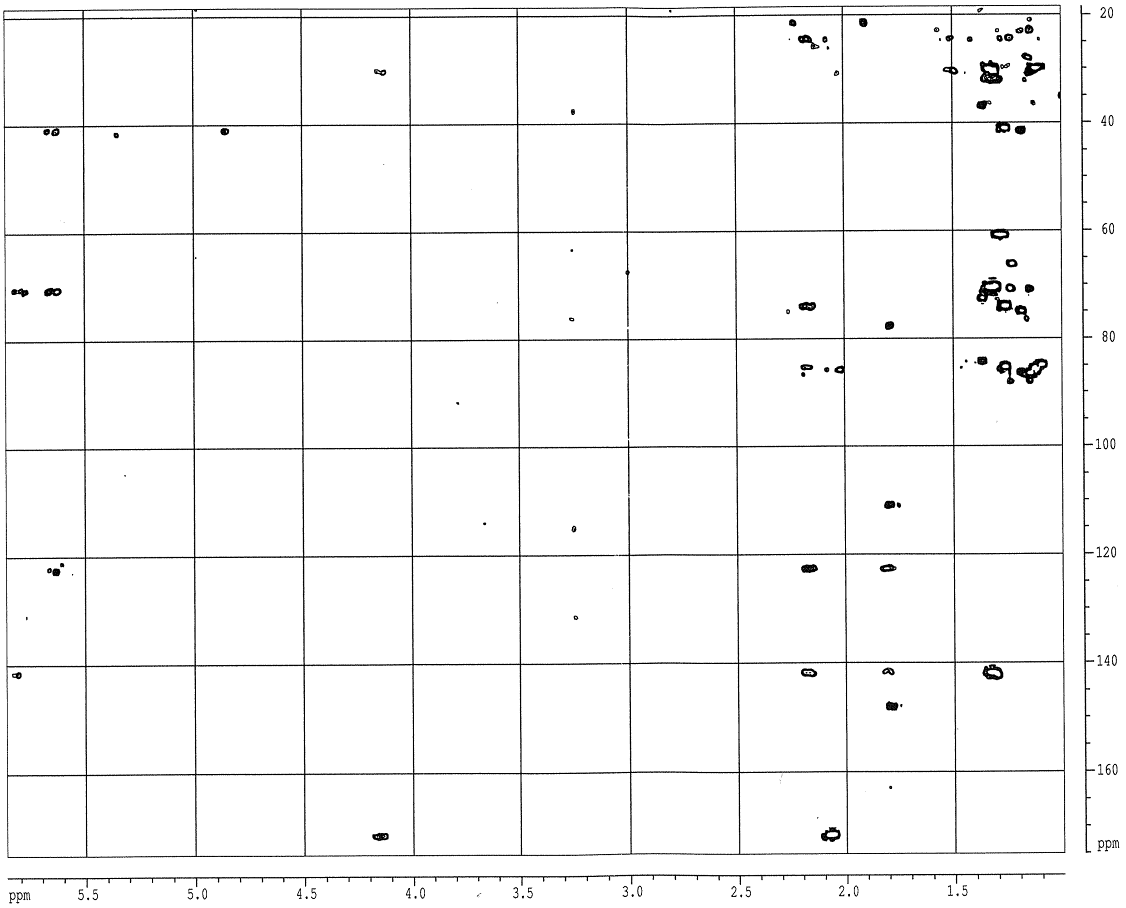

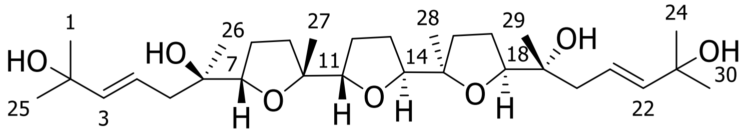

Figure S11. MS spectrum of longilene (**2**).

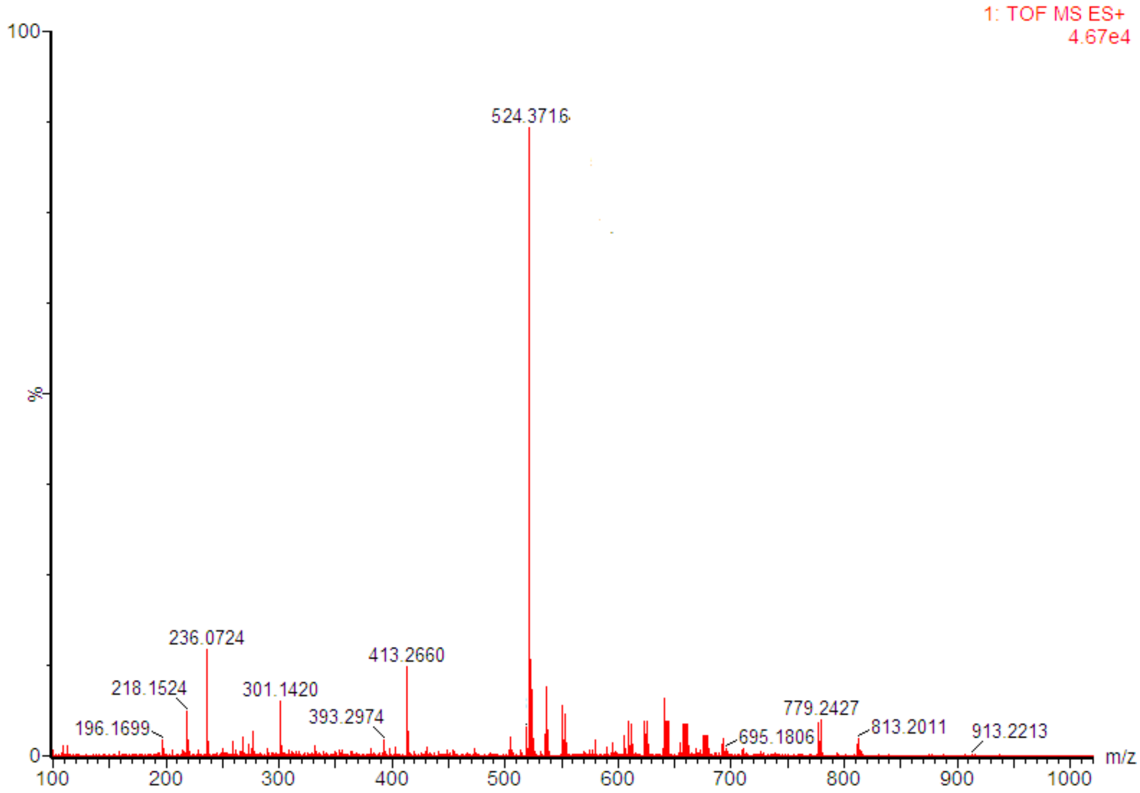

Table S3. NMR data of (+)-prelongilene (**3**) in $CDCl_3$ at 300 K, 500 MHz.

Carbon	δ ¹³C	Multiplicity	δ ¹H	*J* in Hz
**1**	17.7	CH_3	1.60	s
**2**	131.1	C		
**3**	124.8	CH	5.08	t 6.9
**4**	22.5	CH_2	1.97<br>2.04	
**5**	39.0	CH_2	1.29<br>1.43	
**6**	72.5	C		
**7**	83.4	CH	3.73	dd 6.9, 7.6
**8**	25.4	CH_2	1.80<br>1.91	
**9**	30.0	CH_2	1.46<br>2.04	
**10**	84.7	C		
**11**	85.0	CH	4.07	dd 5.4, 10.3
**12**	29.5	CH_2	1.50<br>1.99	
**13**	29.5	CH_2	1.50<br>1.99	
**14**	85.7	CH	4.13	dd 5.7, 10.3
**15**	85.8	C		
**16**	30.0	CH_2	1.46<br>2.06	
**17**	25.5	CH_2	1.93<br>2.14	
**18**	85.0	CH	3.81	dd 4.4, 8.1
**19**	74.1	C		
**20**	40.8	CH_2	1.83<br>2.17	
**21**	122.7	CH	5.74	ddd 6.2, 8.6, 15.2
**22**	141.0	CH	5.62	d 15.2
**23**	70.3	C		
**24**	29.8	CH_3	1.31	s
**25**	25.7	CH_3	1.66	s
**26**	24.9	CH_3	1.27	s
**27**	23.5	CH_3	1.08	s
**28**	24.0	CH_3	1.11	s
**29**	24.1	CH_3	1.20	s
**30**	30.1	CH_3	1.31	s
**-OH-6**			4.94	s
**-OH-19**			4.39	s
**-OH-23**			2.57	s

Figure S12. ^{1}H-NMR spectrum of (+)-prelongilene (**3**) in $CDCl_3$ at 300 K, 500 MHz.

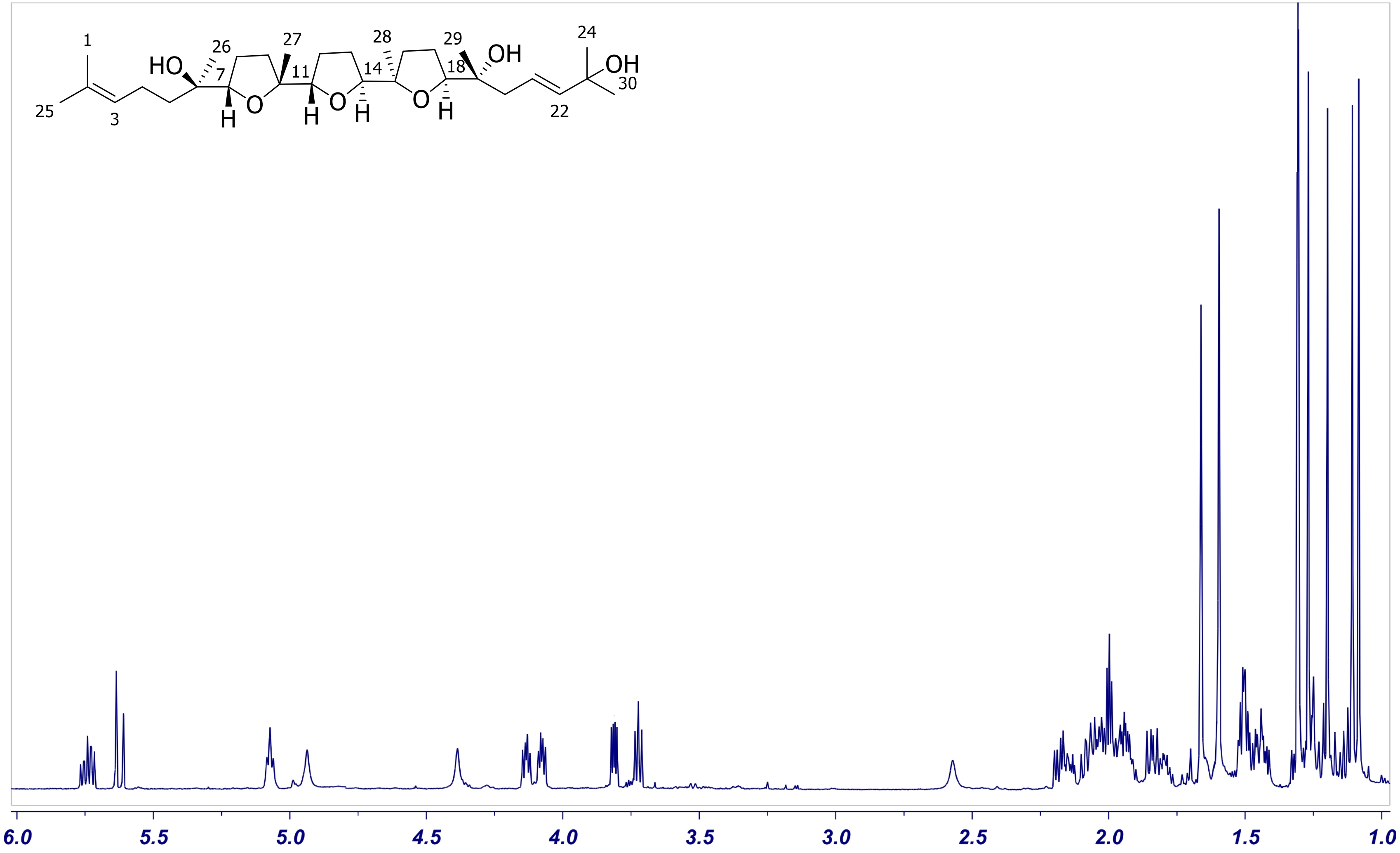

Figure S13. COSY spectrum of (+)-prelongilene (**3**) in $CDCl_3$ at 300 K, 500 MHz.

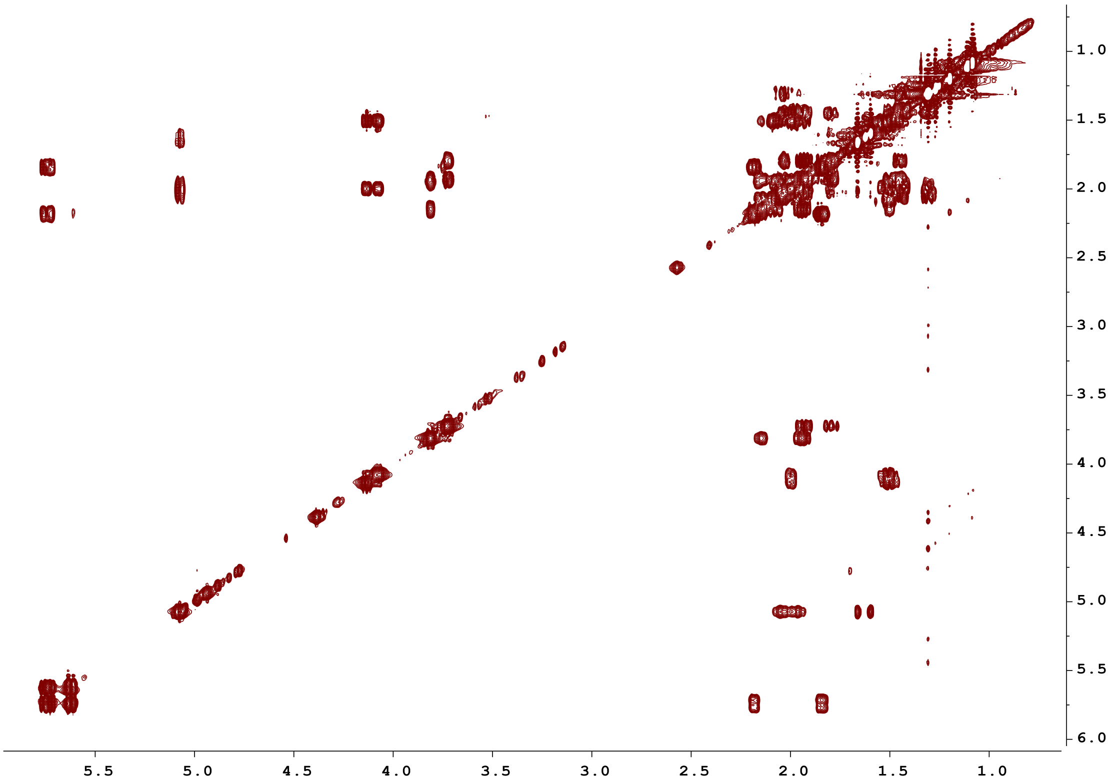

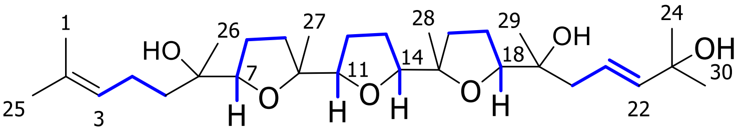

Figure S14. HSQCed spectrum of (+)-prelongilene (**3**) in $CDCl_3$ at 300 K, 500 MHz.

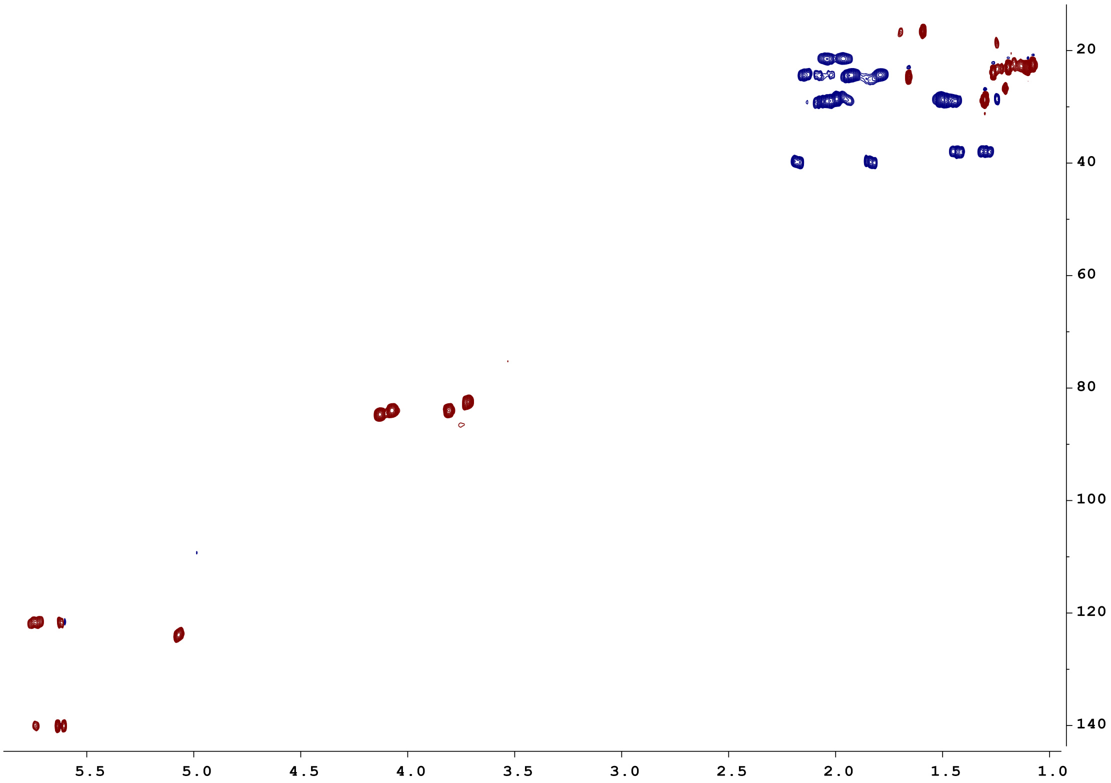

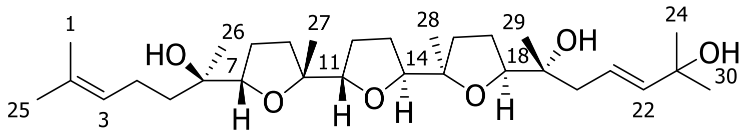

Figure S15. HMBC spectrum of (+)-prelongilene (**3**) in $CDCl_3$ at 300 K, 500 MHz.

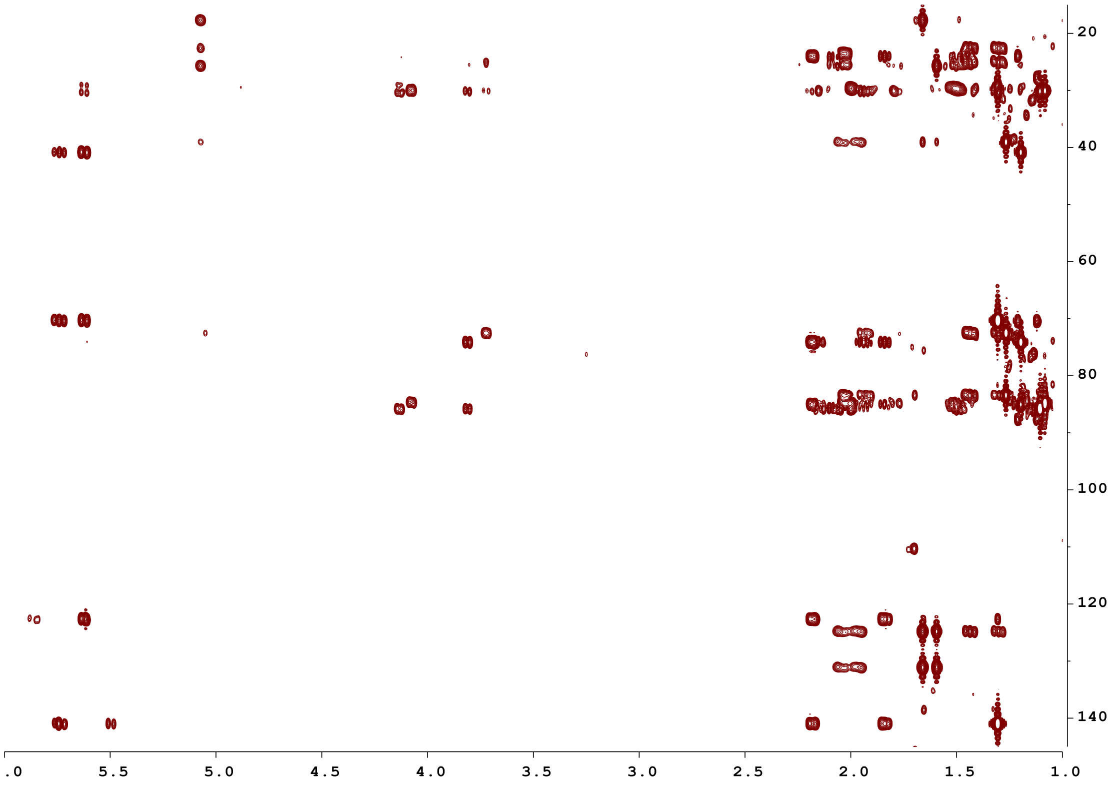

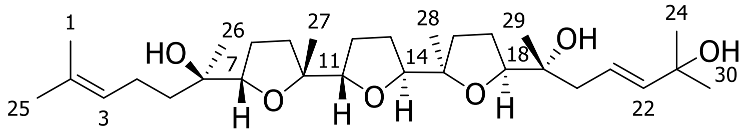

Figure S16. ROESY spectrum of (+)-prelongilene (**3**) in $CDCl_3$ at 300 K, 500 MHz.

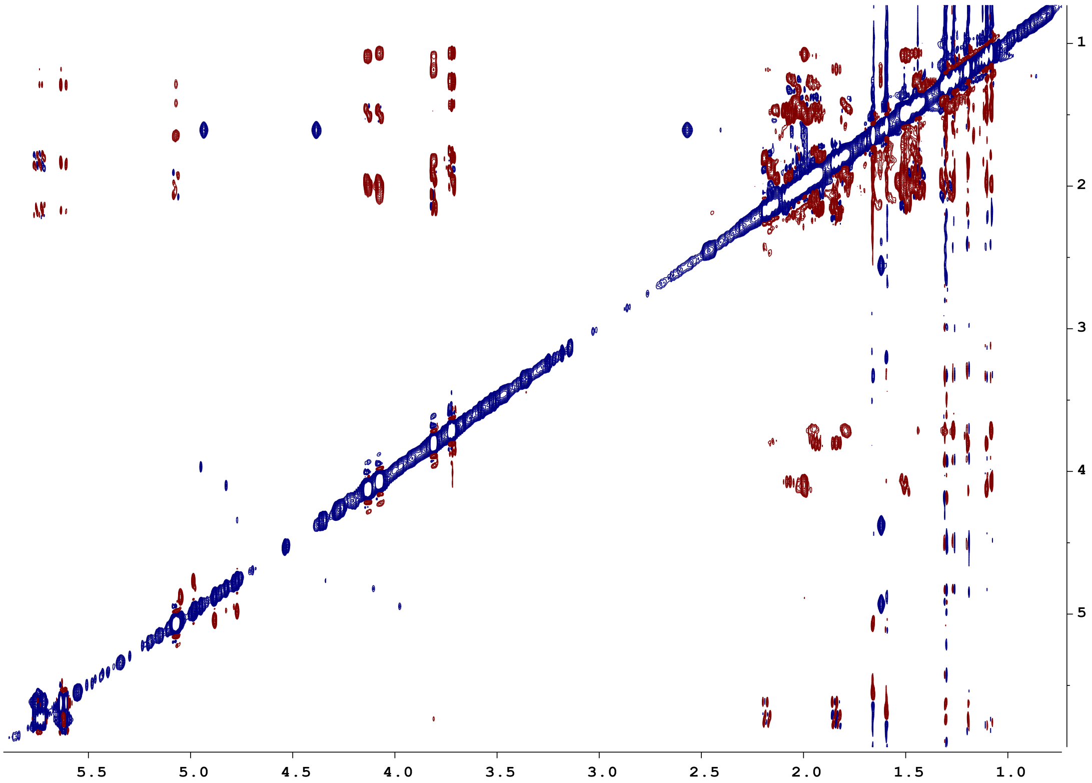

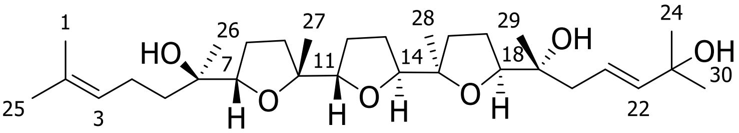

Figure S17. MS spectrum of (+)-prelongilene (**3**).

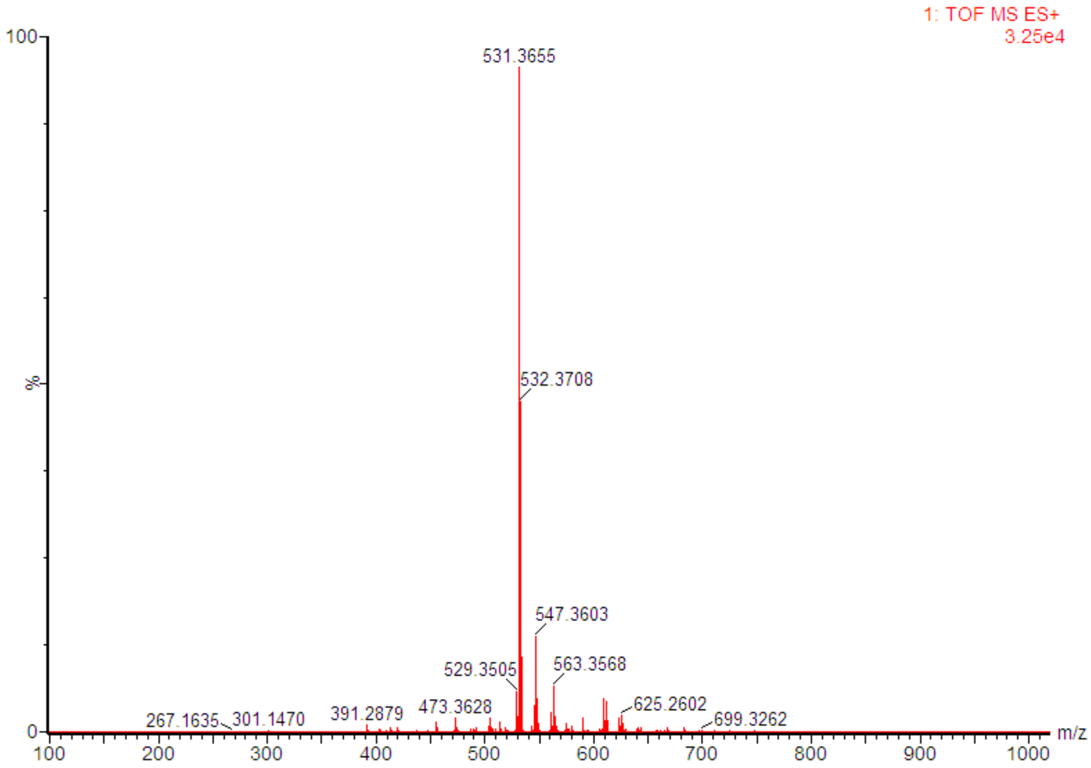

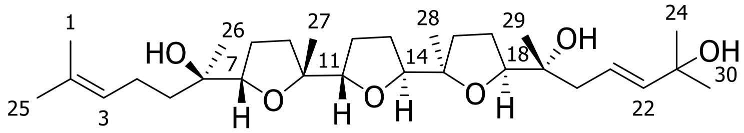

Figure S18. Conversion of (+)-prelongilene (**3**) to compound **5** for ¹H NMR; 0 h.

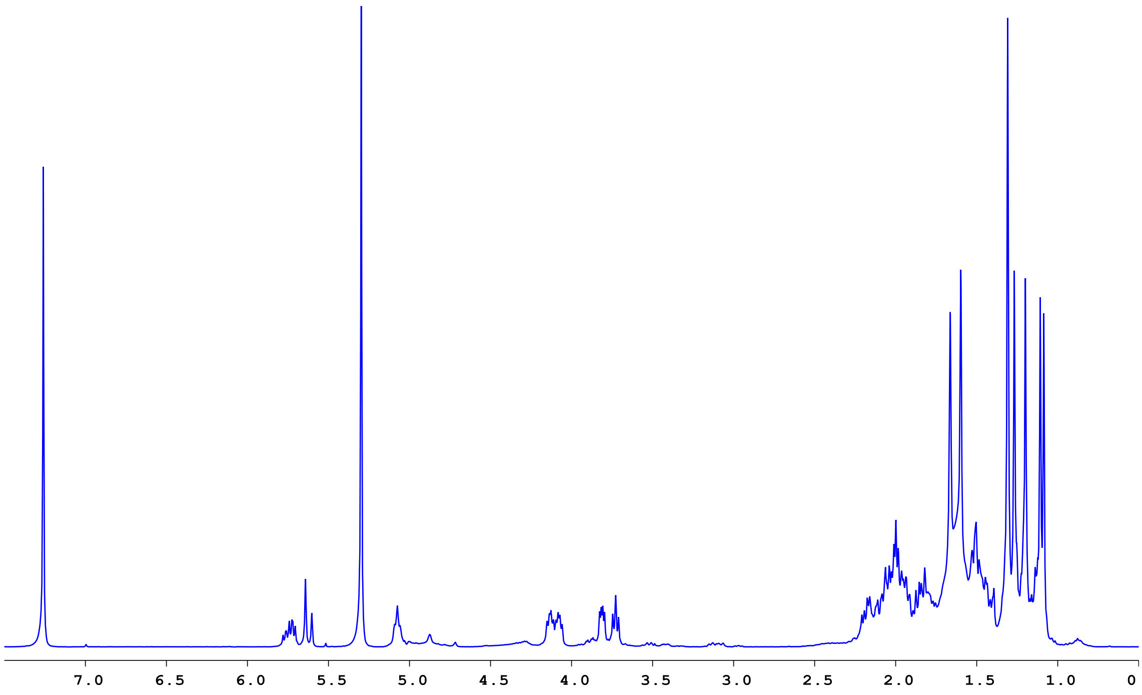

Figure S19. Conversion of (+)-prelongilene (**3**) to compound **5** for ¹H NMR; 24 h.

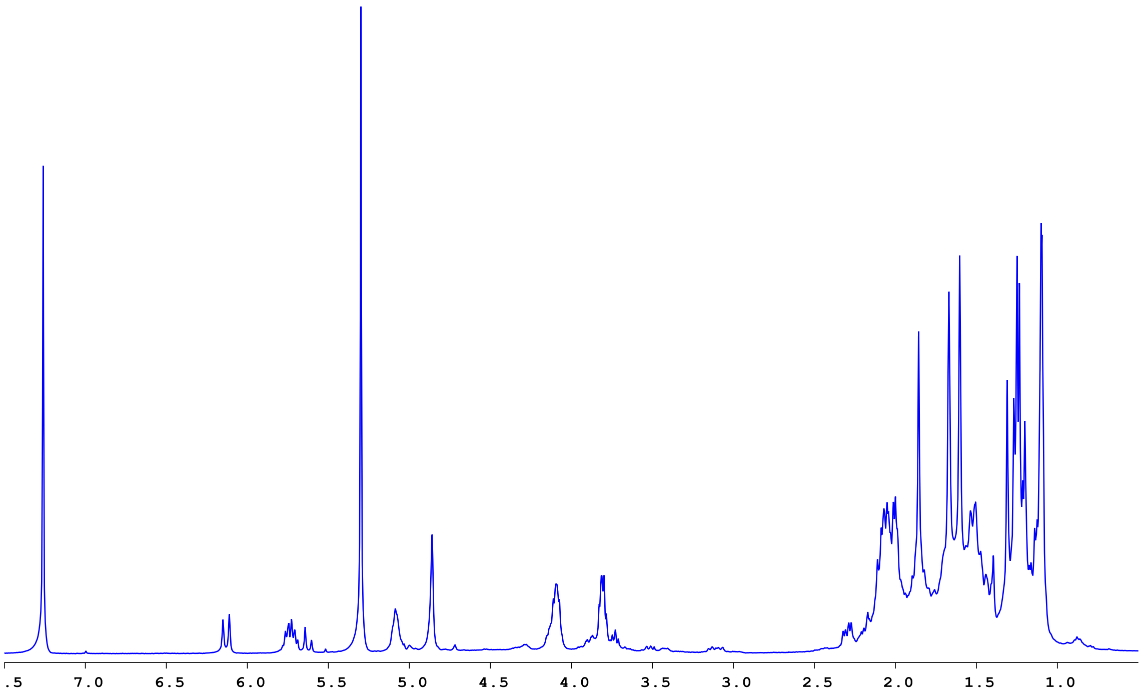

Figure S20. Conversion of (+)-prelongilene (**3**) to compound **5** for ^{1}H NMR; 48 h.

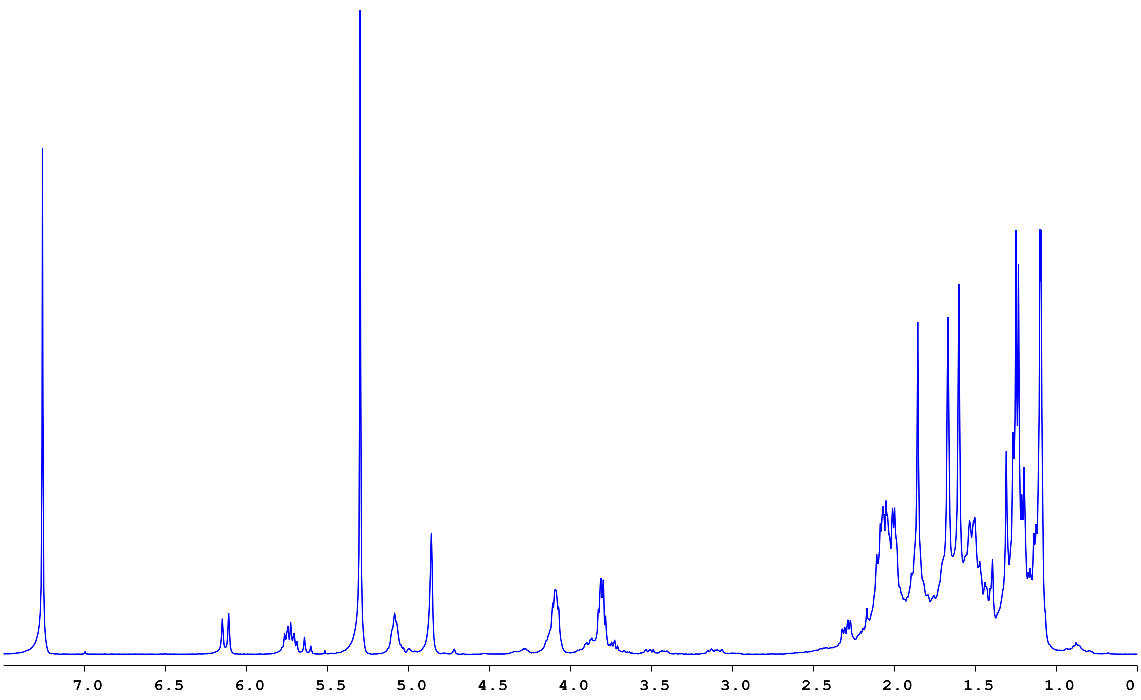

Figure S21. Conversion of (+)-prelongilene (**3**) to compound **5** for ^{1}H NMR; 72 h.

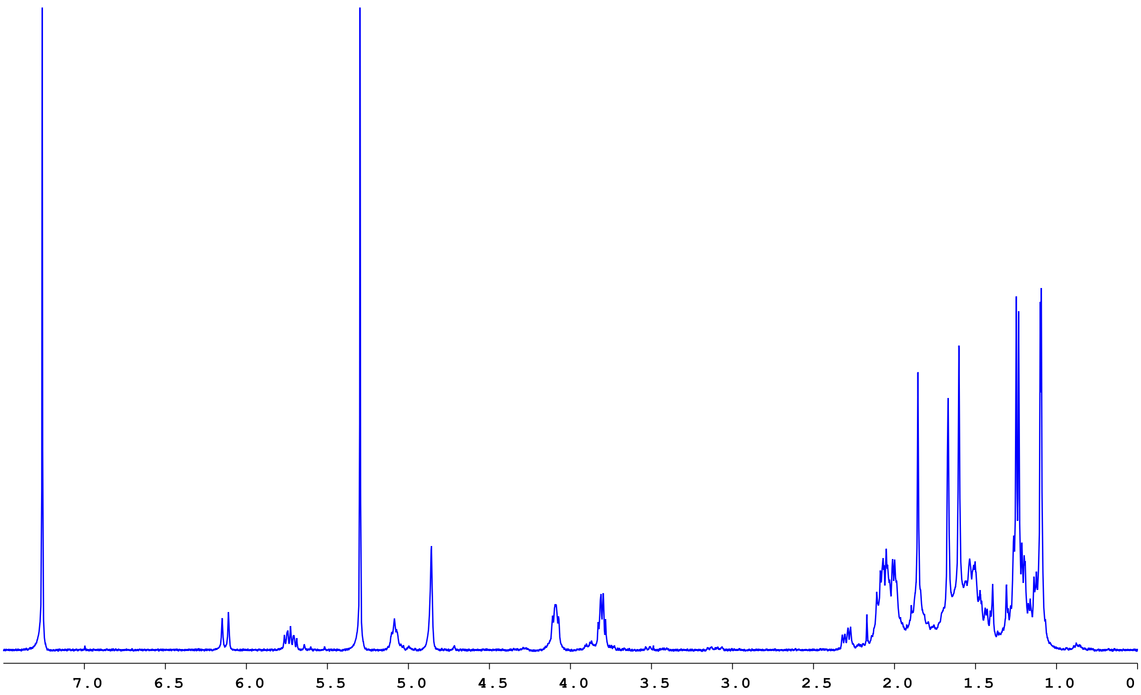

Table S4. NMR data of compound **5** in $CDCl_3$ at 300 K, 500 MHz.

Carbon	δ [13]C	Multiplicity	δ [1]H	*J* in Hz
**1**	17.7	CH_3	1.60	s
**2**	131.2	C		
**3**	124.9	CH	5.08	t 6.8
**4**	22.2	CH_2	1.99<br>2.09	
**5**	38.6	CH_2	1.27<br>1.43	
**6**	73.0	C		
**7**	84.1	CH	3.81	dd 6.5, 11.7
**8**	25.3	CH_2	1.87<br>2.06	
**9**	30.4	CH_2	1.50<br>2.04	
**10**	85.3	C		
**11**	85.2	CH	4.09	dd 5.2, 9.3
**12**	29.5	CH_2	1.53<br>2.03	
**13**	29.5	CH_2	1.53<br>2.03	
**14**	85.2	CH	4.09	dd 5.2, 9.3
**15**	85.4	C		
**16**	30.4	CH_2	1.50<br>2.04	
**17**	25.3	CH_2	1.85<br>2.06	
**18**	84.1	CH	3.81	dd 6.5, 11.7
**19**	73.4	C		
**20**	42.5	CH_2	2.09<br>2.29	
**21**	126.4	CH	5.72	ddd 6.3, 8.7, 15.3
**22**	135.1	CH	6.13	d 15.6
**23**	142.3	C		
**24**	114.4	CH	4.86	s
**25**	25.7	CH_3	1.67	s
**26**	24.3	CH_3	1.23	s
**27**	23.5	CH_3	1.09	s
**28**	23.6	CH_3	1.10	s
**29**	24.3	CH_3	1.25	s
**30**	18.8	CH_3	1.86	s

Figure S22. ^{1}H-NMR spectrum of compound **5** in $CDCl_3$ at 300 K, 500 MHz.

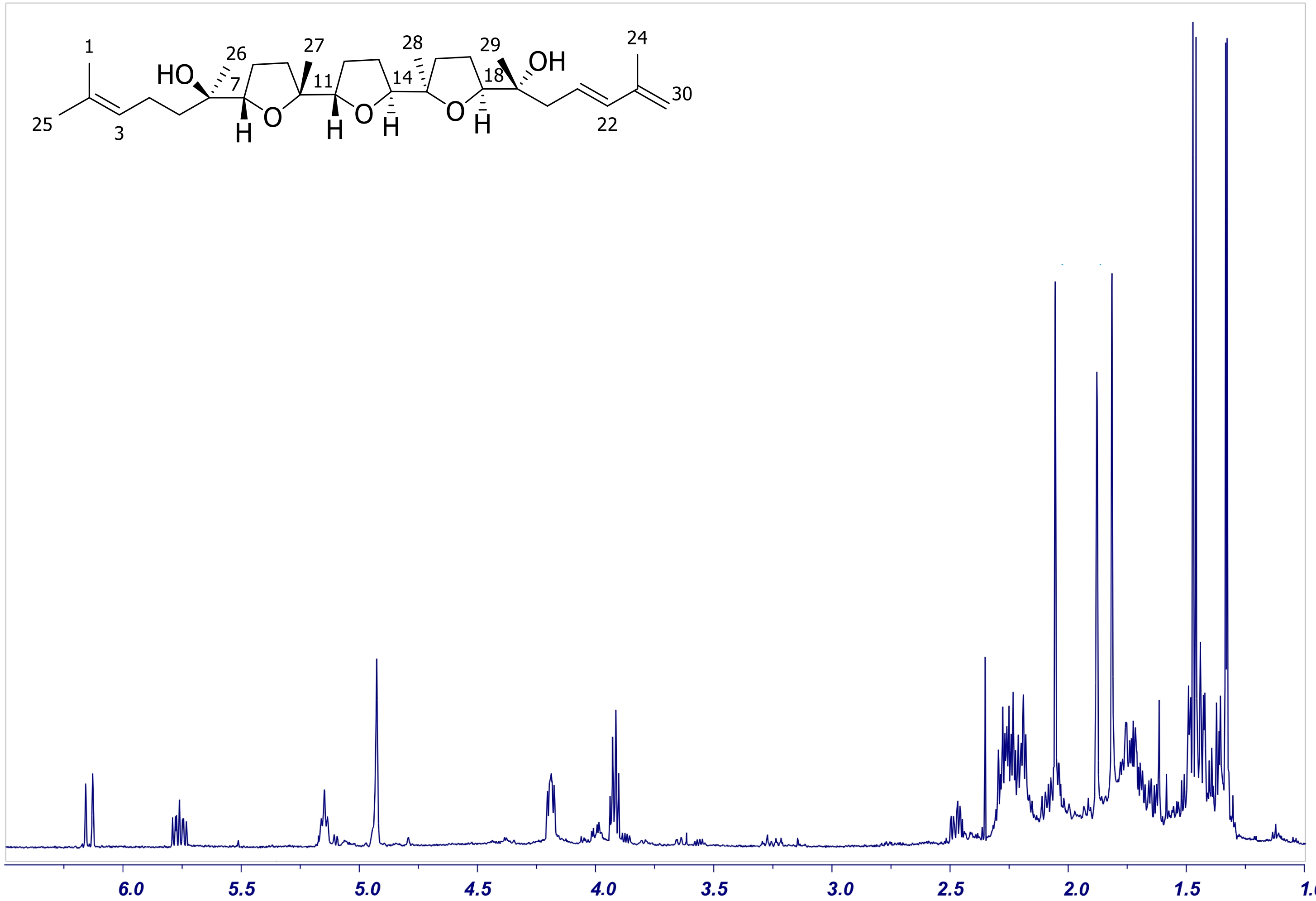

Figure S23. COSY spectrum of compound **5** in $CDCl_3$ at 300 K, 500 MHz.

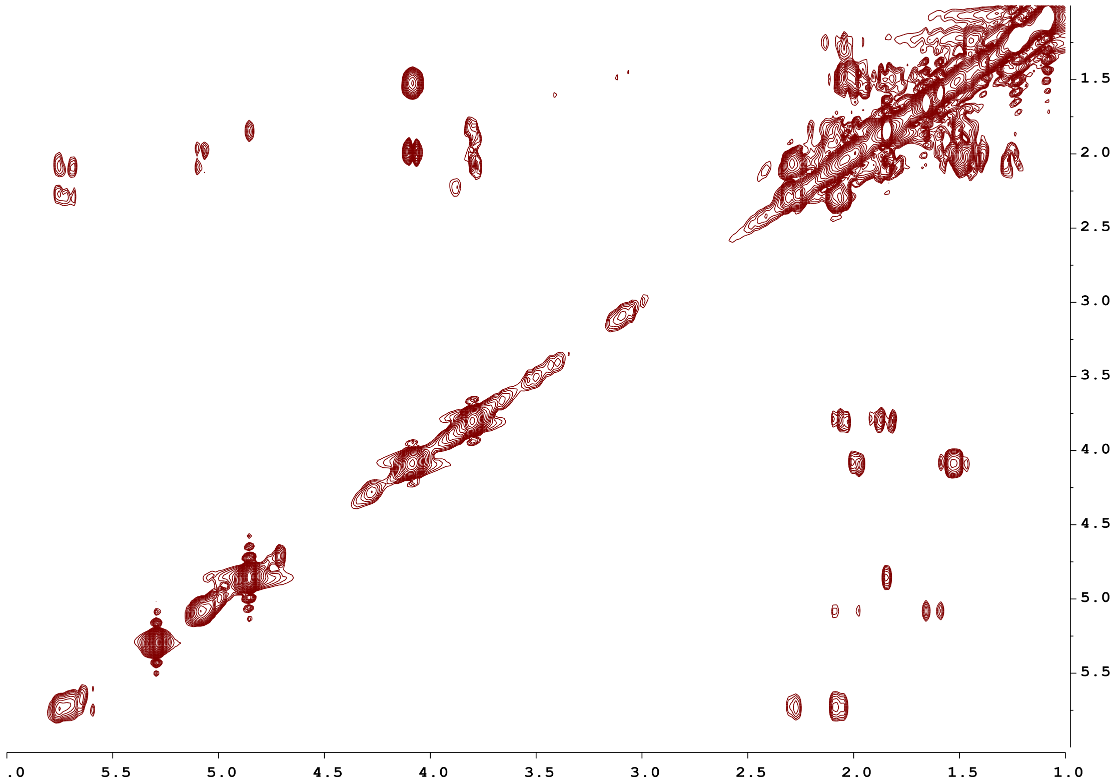

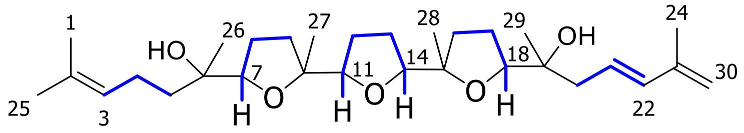

Figure S24. HSQC spectrum of compound **5** in $CDCl_3$ at 300 K, 500 MHz.

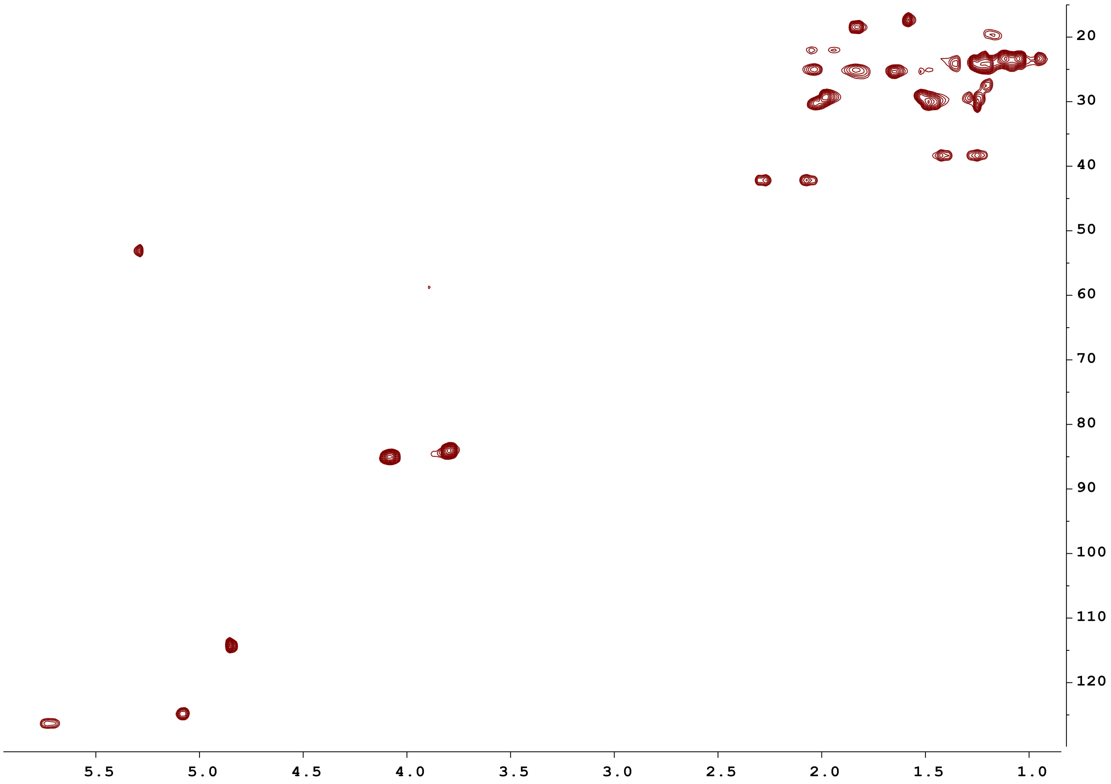

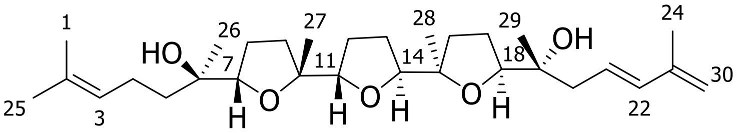

Figure S25. HMBC spectrum of compound **5** in $CDCl_3$ at 300 K, 500 MHz.

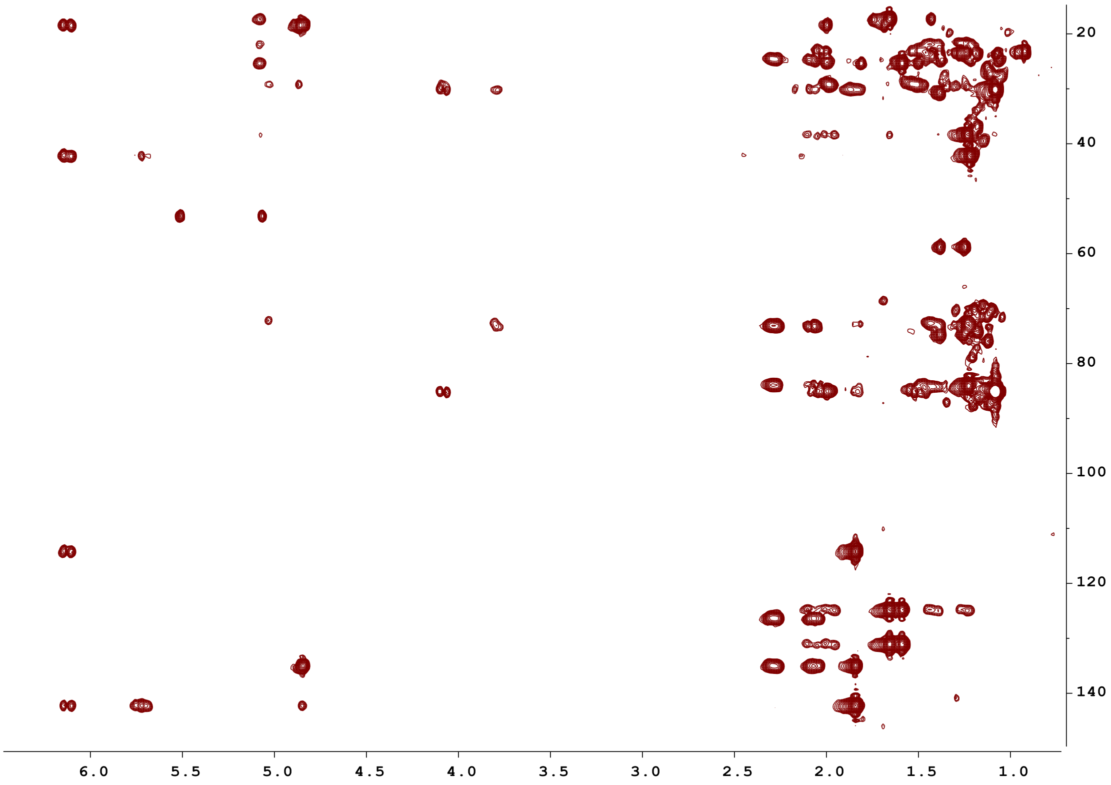

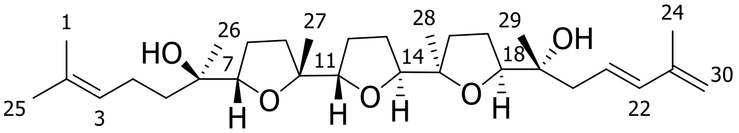

Figure S26. NOESY spectrum of compound **5** in $CDCl_3$ at 300 K, 500 MHz.

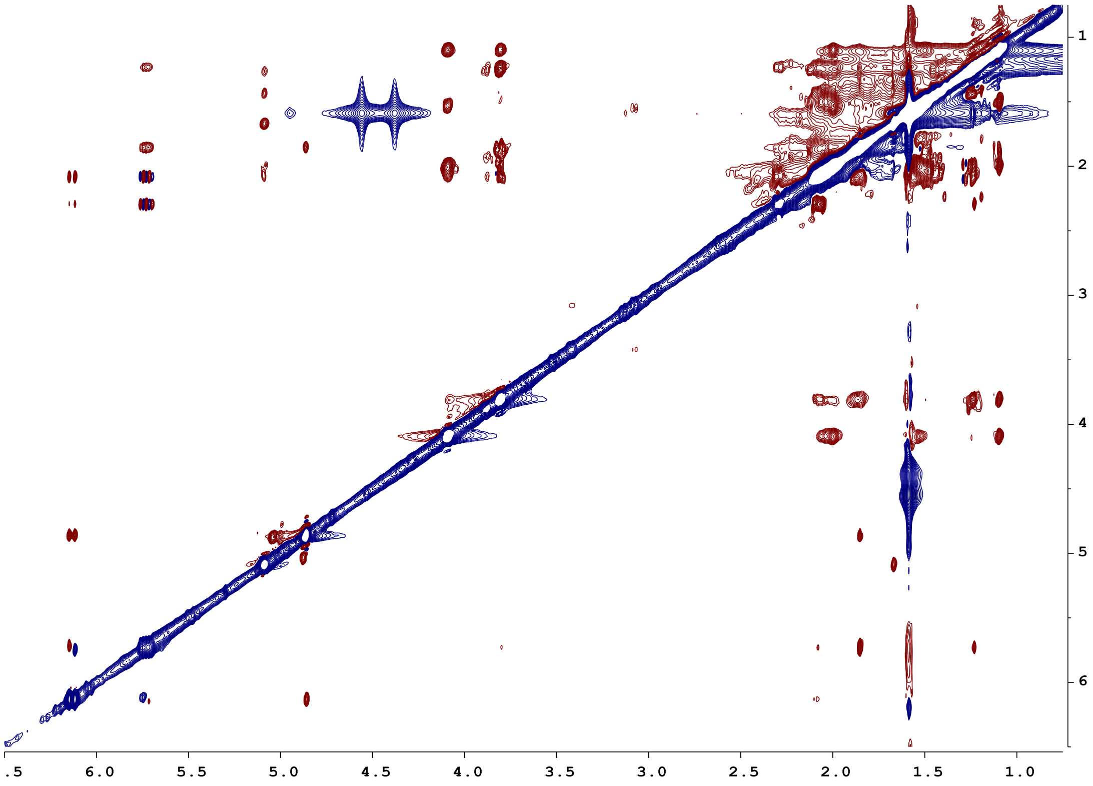

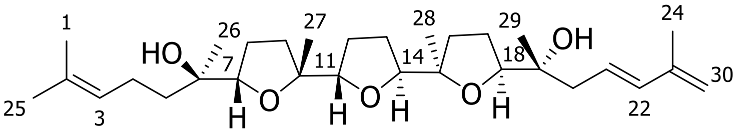

Figure S27. MS spectrum of compound **5**.

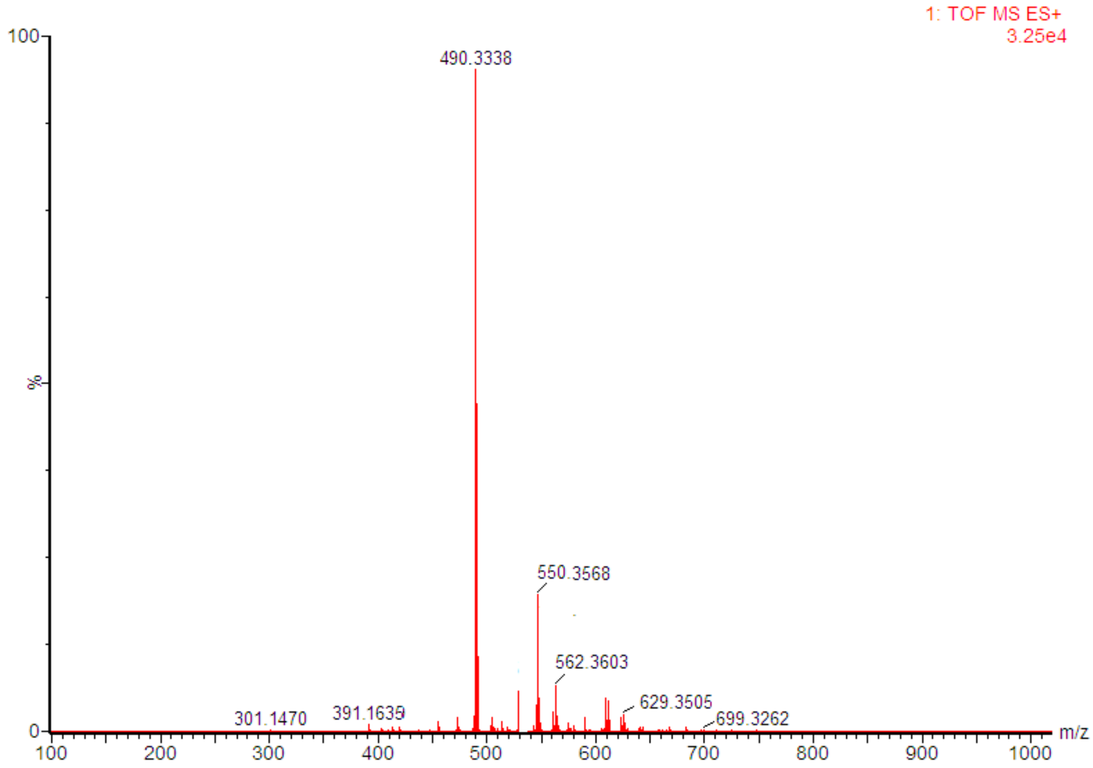

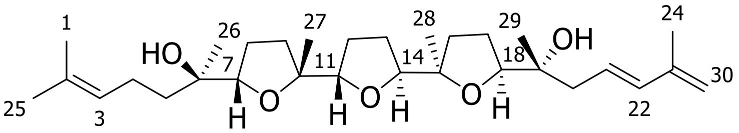

Table S5. NMR data of compound **4** in $CDCl_3$ at 300 K, 500 MHz.

Carbon		δ ^{13}C	Multiplicity	δ ^{1}H	*J* in Hz
**1**	**24**	18.4	CH_3	1.85	s
**2**	**23**	142.3	C		
**3**	**22**	135.2	CH	6.13	d 15.6
**4**	**21**	126.2	CH	5.72	ddd 7.2, 7.7, 15.6
**5**	**20**	42.3	CH_2	2.07<br>2.29	dd 7.2, 13.1<br>dd 7.7, 13.1
**6**	**19**	73.5	C		
**7**	**18**	84.0	CH	3.82	dd 5.9, 6.5
**8**	**17**	25.2	CH_2	1.88<br>2.09	
**9**	**16**	30.4	CH_2	1.52<br>2.04	
**10**	**15**	85.7	C		
**11**	**14**	85.4	CH	4.10	dd 5.4, 8.7
**12**	**13**	29.2	CH_2	1.49<br>2.01	
**25**	**30**	114.5	CH_3	4.86	bs
**26**	**29**	24.4	CH_3	1.24	s
**27**	**28**	23.3	CH_3	1.10	s

Figure S28. ^{1}H-NMR spectrum of compound **4** in $CDCl_3$ at 300 K, 500 MHz.

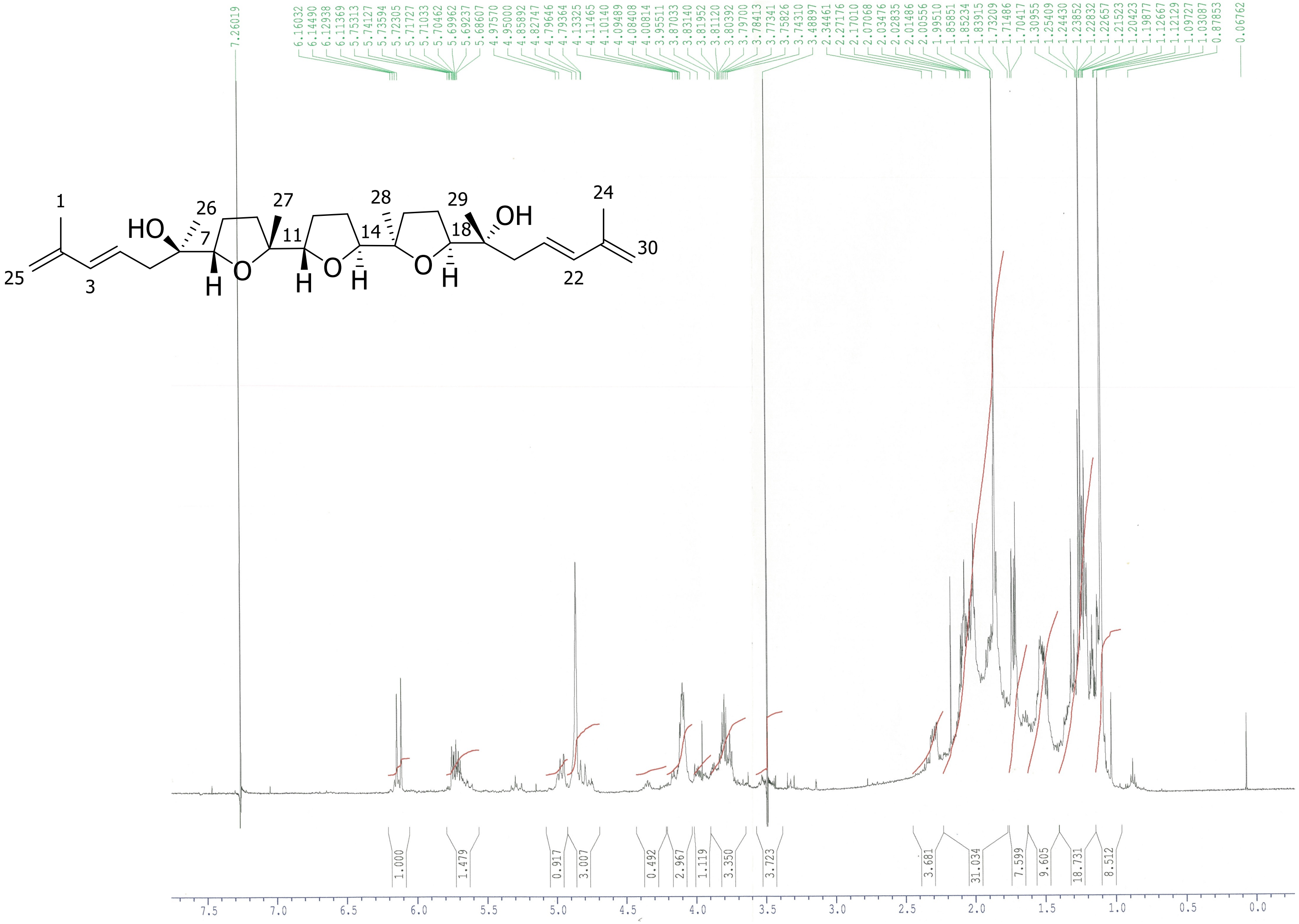

Figure S29. HSQC spectrum of compound **4** in $CDCl_3$ at 300 K, 500 MHz.

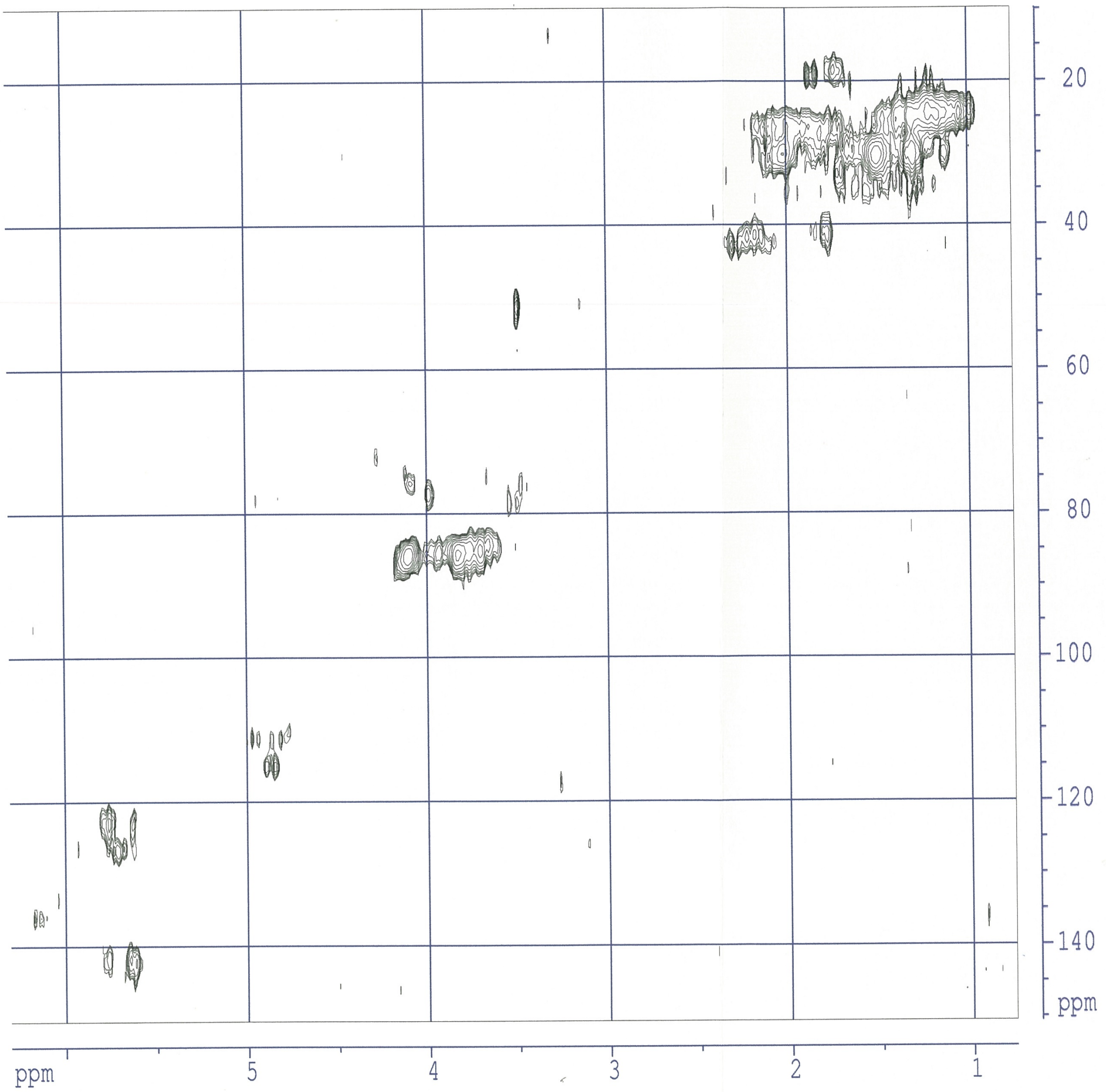

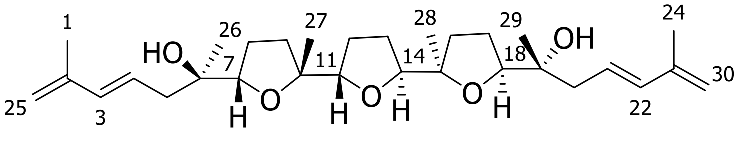

Figure S30. HMBC spectrum of compound **4** in $CDCl_3$ at 300 K, 500 MHz.

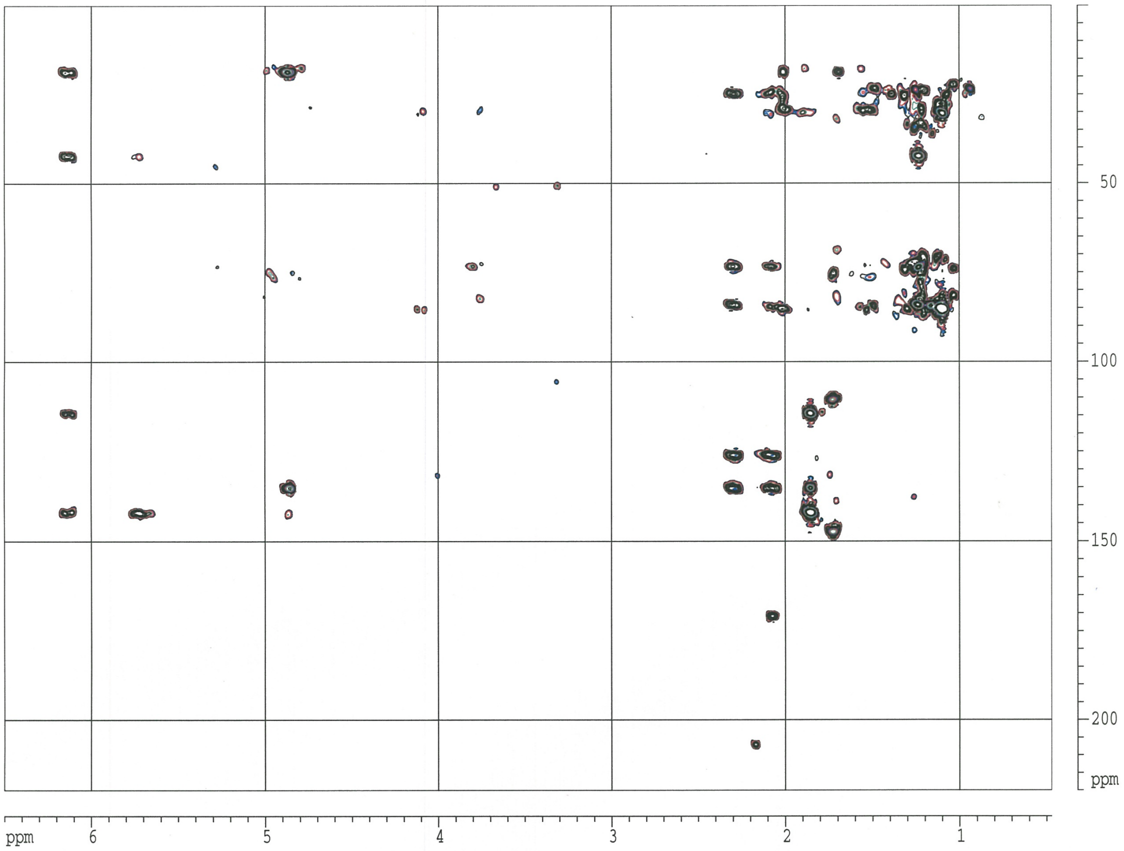

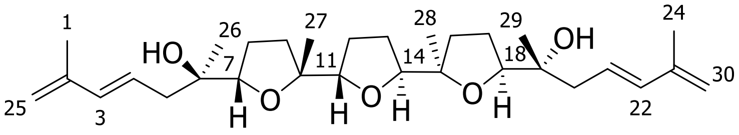

Figure S31. NOESY spectrum of compound **4** in $CDCl_3$ at 300 K, 500 MHz.

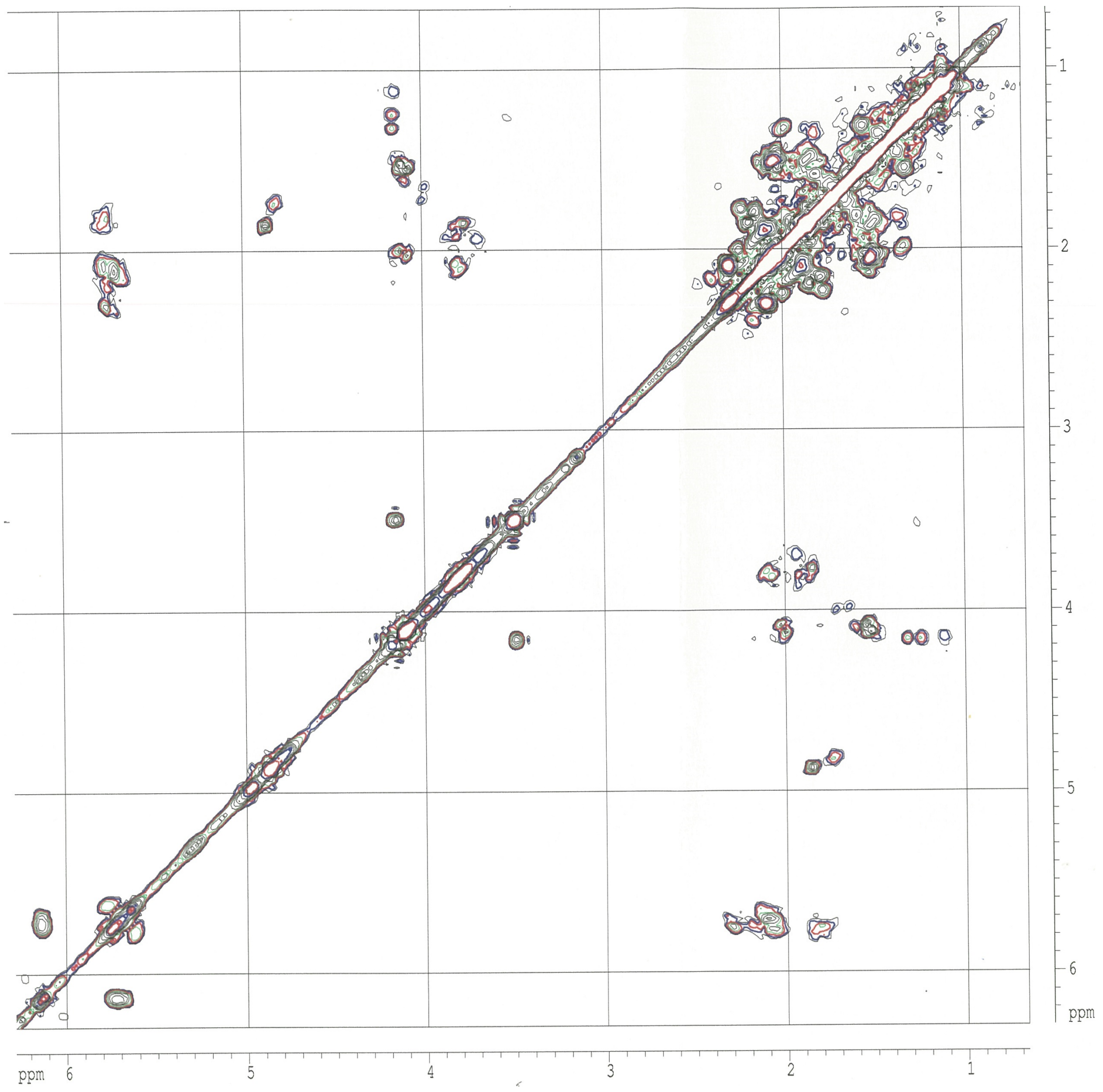

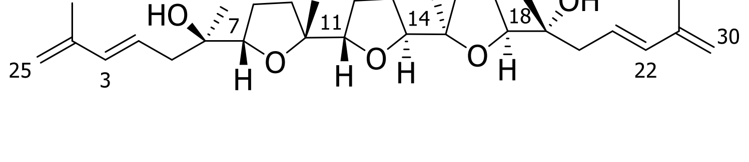

Figure S32. *In vitro* inhibitory effect of (+)-longilene peroxide (**1**) on PP2A

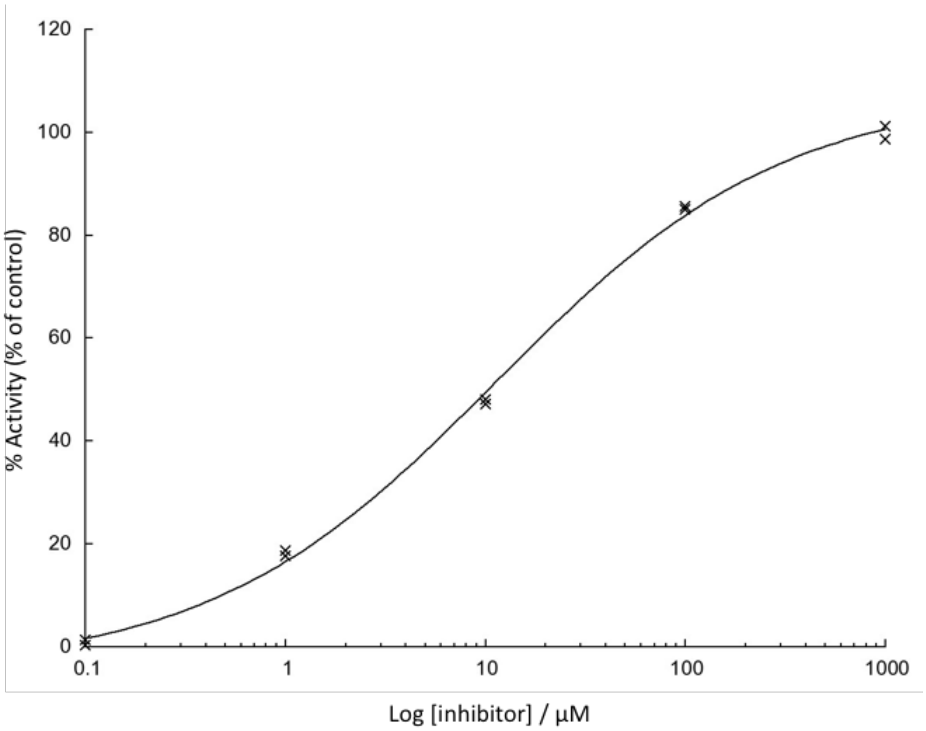

Supplement: Supplementary file 1 [file marinedrugs-16-00131-s001.pdf]
